# Notch signaling pathway: a comprehensive prognostic and gene expression profile analysis in breast cancer

**DOI:** 10.1186/s12885-022-10383-z

**Published:** 2022-12-07

**Authors:** Hassan Yousefi, Afshin Bahramy, Narges Zafari, Mahsa Rostamian Delavar, Khoa Nguyen, Atousa Haghi, Tahmineh Kandelouei, Cecilia Vittori, Parham Jazireian, Sajad Maleki, Danyal Imani, Amin Moshksar, Amirreza Bitaraf, Sadegh Babashah

**Affiliations:** 1 Biochemistry & Molecular Biology, Louisiana State University Health Science Center (LSUHSC), New Orleans, LA USA; 2 Department of Medical Genetics, Faculty of Medical Sciences, Tarbiat Modares University, Tehran, Iran; 3 Department of Medical Genetics, School of Medicine, Tehran University of Medical Sciences, Tehran, Iran; 4 Department of Cell and Molecular Biology and Microbiology, Faculty of Biological Science and Technology, University of Isfahan, Isfahan, Iran; 5 Department of Medicine, Tulane University School of Medicine, New Orleans, LA USA; 6 Hematology Oncology and Stem Cell Transplantation Research Center, Tehran University of Medical Sciences, Tehran, Iran; 7 Huntsman Cancer Institute, University of Utah School of Medicine, Salt Lake City, Utah USA; 8 Louisiana State University Health Sciences Center (LSUHSC), and Stanley S. Scott Cancer Center, New Orleans, LA USA; 9 Department of Biology, University Campus 2, University of Guilan, Rasht, Iran; 10 Department of Biochemistry, Faculty of Biological Sciences, Tarbiat Modares University, Tehran, Iran; 11 Department of Immunology, School of Public Health, Tehran University of Medical Sciences (TUMS), Tehran, Iran; 12 Interventional Radiology, University of Texas Medical Branch (UTMB), Galveston, TX USA; 13 Department of Molecular Genetics, Faculty of Biological Sciences, Tarbiat Modares University, P.O. Box, Tehran, 14115-154 Iran

**Keywords:** Breast cancer, NOTCH pathway, Methylation

## Abstract

Breast cancer is a complex disease exhibiting a great degree of heterogeneity due to different molecular subtypes. Notch signaling regulates the differentiation of breast epithelial cells during normal development and plays a crucial role in breast cancer progression through the abnormal expression of the Notch up-and down-stream effectors. To date, there are only a few patient-centered clinical studies using datasets characterizing the role of Notch signaling pathway regulators in breast cancer; thus, we investigate the role and functionality of these factors in different subtypes using publicly available databases containing records from large studies. High-throughput genomic data and clinical information extracted from TCGA were analyzed. We performed Kaplan–Meier survival and differential gene expression analyses using the HALLMARK_NOTCH_SIGNALING gene set. To determine if epigenetic regulation of the Notch regulators contributes to their expression, we analyzed methylation levels of these factors using the TCGA HumanMethylation450 Array data. Notch receptors and ligands expression is generally associated with the tumor subtype, grade, and stage. Furthermore, we showed gene expression levels of most Notch factors were associated with DNA methylation rate. Modulating the expression levels of Notch receptors and effectors can be a potential therapeutic approach for breast cancer. As we outline herein, elucidating the novel prognostic and regulatory roles of Notch implicate this pathway as an essential mediator controlling breast cancer progression.

**Supplementary Information:**

The online version contains supplementary material available at 10.1186/s12885-022-10383-z.

## Introduction

Breast cancer is a heterogeneous disease and is the second cause of cancer death among women globally. Among all women newly diagnosed with cancer, 30% suffered from breast cancer [1, 2]. Breast cancer can be categorized into five distinct molecular subtypes: claudin-low (12–14%), basal-like (15–20%), HER2 enriched (10–15%), luminal A (40%), and luminal B (20%) [3]. Each molecular subtype of breast cancer has unique qualities that affect its treatment strategy and clinical outcomes. For example, luminal breast cancers positively respond to anti-hormonal therapy with a better prognosis. At the same time, basal-like subtypes tend to be treatment-resistant and have the shortest patient survival rates [4, 5]. Additionally, the molecular characteristics of the basal subtype make it resistant to current chemotherapies. Furthermore, there are limited options for targeted therapies. Overall, these qualities make it necessary to find an optimal target to develop treatment strategies for the aggressive basal subtypes. As an evolutionarily conserved pathway, the Notch signaling has fundamental functions as both a suppresser and promoter of proliferation, an activator of differentiation, and is the primary regulator of apoptosis, cell fate, and tissue renewal [6]. Aberrant expression and mutation of the Notch signaling pathway have been established in various diseases such as solid tumors and developmental disorders [7, 8]. The association between breast cancer and the Notch signaling pathway was first noted in mice [9]. Mice infected with MMTV (mouse mammary tumor virus) resulted in a hot spot insertion in the Notch 4 locus, which led to the constitutive expression of NOTCH-4 and its target genes, truncation and activation of NOTCH-1 as an oncogene, and eventual development of mammary adenocarcinoma [10, 11]. High expression of Notch receptors and ligands was reported to be associated with poor survival in breast cancer patients [12, 13]. Several studies have shown that dysregulated expression of NOTCH-1, NOTCH-2, and NOTCH-4 result in oncogenesis. In contrast, NOTCH-3 acts as a tumor suppressor, in human breast cancer, with mutations resulting in elevated tumor proliferation, invasion, angiogenesis, epithelial-to-mesenchymal transition (EMT), and self-renewal of breast cancer stem-like cells [12, 14–20]. Another study showed that upregulation of NOTCH-1, NOTCH-3, and Jagged1 positively correlates with a mortality rate [12]. Altogether, the existing evidence suggests that the Notch pathway plays multiple critical roles in breast cancer progression.

Dysregulation of the Notch signaling pathway might have different results depending on the breast cancer subtype. This is due to the different gene expression profiles and unique disease prognosis of each subtype. For instance, an increased expression of NOTCH-3 receptor was found in Luminal A. Increased NOTCH-4 was found in both Luminal A and B of breast cancer [21, 22]. On the other hand, in the basal-like/triple-negative breast cancer (TNBC) subtype, higher activity of NOTCH-1, NOTCH-3, NOTCH-4, and Jagged-1 were reported, which increased angiogenesis and are associated with poor clinical prognosis of patients [23–25]. The Notch signaling pathway promotes tumor progression and survival and induces breast cancer stem cells (CSCs) phenotype [26]. The association between dysregulation of Notch signaling and overall survival rate in breast cancer patients is a useful tool to exploit as prognostic biomarkers. For example, it has been shown that NOTCH-1 high-expressing women with breast cancer had a 66% mortality rate compared to 30.5% for patients with low expression levels. Accordingly, the mortality rate in patients with upregulation of Jagged-1 was 63% compared to 32% in patients who had low expression of Jagged-1 [12]. Furthermore, tumor grade and NOTCH-1 have shown to be positively correlated [27]. In this study, we analyzed the expression pattern of Notch signaling receptors to understand how these critical tumorigenic factor genes are expressed in breast cancer subtypes, and we examined how their associated effector genes are expressed in the subtypes. We then evaluated whether these gene expression profiles can predict the survival rates within each breast cancer subtype or how their expression might be associated with tumor grade using high-throughput genomic data of primary tumors from the Cancer Genome Atlas (TCGA) [28]. Numerous studies have explored non-coding RNAs' role in breast cancer's progression and molecular mechanisms so far [29, 30]. In addition, many studies have shown that the Notch-Signaling pathway is one of the critical pathways in breast cancer [31]. Also, in recent years, researchers have done many experimental and functional studies to confirm the role of various non-coding RNAs, including miRNAs and LncRNAs, in the Notch-signaling pathway regulation [32]. Accordingly, as previous studies have not dealt with finding the mechanisms that ncRNAs through which interact with the Notch signaling pathway in the context of breast cancer, we aimed to conduct an in-silico study to investigate the relationship between non-coding RNAs and Notch signaling pathway-related genes in breast cancer to emphasis on how these non-coding RNAs (miRNAs, LncRNAs) might potentially interact with the Notch signaling pathway using gene ontology analysis. Accordingly, our analysis showed the potential non-coding RNAs regulatory network that might interact with the Notch pathway. Due to its critical role in breast cancer progression, Notch receptors and downstream factors can serve as potential targets in breast cancer therapy. The specific expression profile of each element in an individual tumor would provide the most promising therapeutic target in that subtype. Therefore, specific treatment approaches targeting a common factor in a particular subtype might have the most significant potential for translation to the clinic for breast cancer patients.

## Results

### Notch receptors (NOTCH-1, NOTCH-2, and NOTCH-3) are highly expressed in basal/claudin-low breast cancer subtypes.

**Fig. 1:**
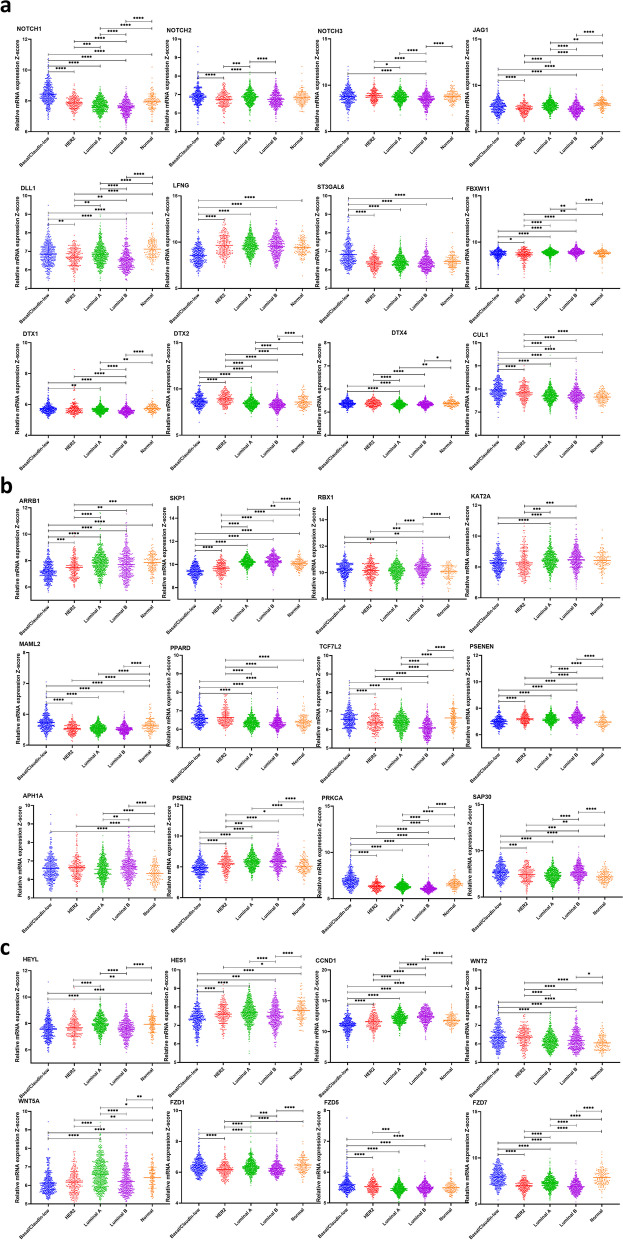
**a** Differential expression of the Notch signaling pathway genes in different breast cancer subtypes. Gene expression of the Notch receptors NOTCH1, NOTCH2, NOTCH3; Notch Ligands JAG1, DLL1, and LFNG, ST3GAL6, FBXW11, DTX1, DTX2, DTX4, and CUL1. Data were extracted from the METABRIC dataset and categorized into five subtypes according to Pam50 gene expression subtype classification (Basal/Claudin-low, HER2, Luminal A, and Luminal B). Scatterplots show a significant association between breast cancer subtypes and the level of gene expression in breast cancer patients. Data were analyzed by one-way ANOVA followed by Tukey’s post hoc test. Statistically significant values of (P-value*: P-value < 0.05, **: P-value < 0.001, ***: 0.0001 < P-value = 0.0001, ****: P-value < 0.0001) were determined. **b** Differential expression of the Notch signaling pathway genes in different breast cancer subtypes. ARRB1, SKP1, RBX1, KAT2A, MAML2, PPARD, TCF7L2, PSENEN, APHIA, PSEN2, PRKCA, and SAP30 gene expression pattern in breast cancer subtypes. Data were extracted from the METABRIC dataset and categorized into five subtypes according to Pam50 gene expression subtype classification (Basal/Claudin-low, HER2, Luminal A, and Luminal B). Scatterplots show a significant association between breast cancer subtypes and the level of gene expression in breast cancer patients. Data were analyzed by one-way ANOVA followed by Tukey’s post hoc test. Statistically significant values of (P-value*: P-value < 0.05, **: P-value < 0.001, ***: 0.0001 < P-value = 0.0001, ****: P-value < 0.0001) were determined. **c** Differential expression of the Notch signaling pathway genes in different breast cancer subtypes. HEYL, HES1, CCND1, WNT2, WNT5A, FZD1, FZD5, and FZD7 gene expression in different breast cancer subtypes. Data were extracted from the METABRIC dataset and categorized into five subtypes according to Pam50 gene expression subtype classification (Basal/Claudin-low, HER2, Luminal A, and Luminal B). Scatterplots show a significant association between breast cancer subtypes and the level of gene expression in breast cancer patients. Data were analyzed by one-way ANOVA followed by Tukey’s post hoc test. Statistically significant values of (P-value*: P-value < 0.05, **: P-value < 0.001, ***: 0.0001 < P-value = 0.0001, ****: P-value < 0.0001) were determined

**Fig. 2:**
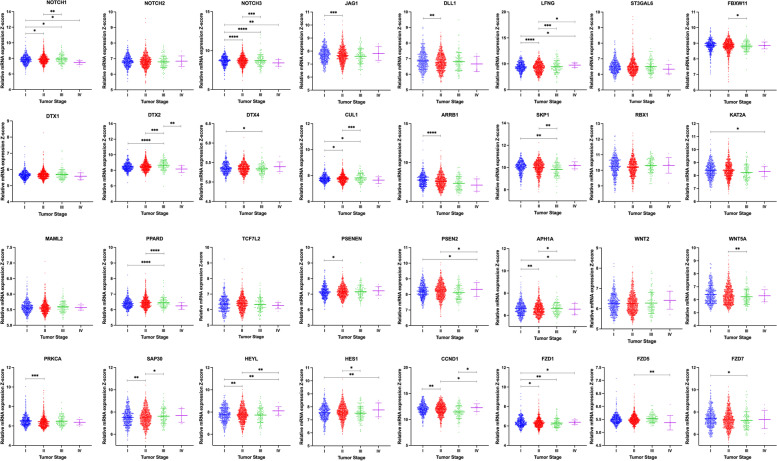
Relative expression of Notch signaling genes in different breast cancer tumor stages. Data were extracted from the METABRIC dataset and analyzed by one-way ANOVA followed by Tukey’s post hoc test. Tumor stage (I, *n* = 475; II, *n* = 800; III, *n* = 115; IV, *n* = 9). Statistically significant values of (*P*-value*: *P*-value < 0.05, **: *P*-value < 0.001, ***: 0.0001 < *P*-value = 0.0001, ****: *P*-value < 0.0001) were determined

**Fig. 3:**
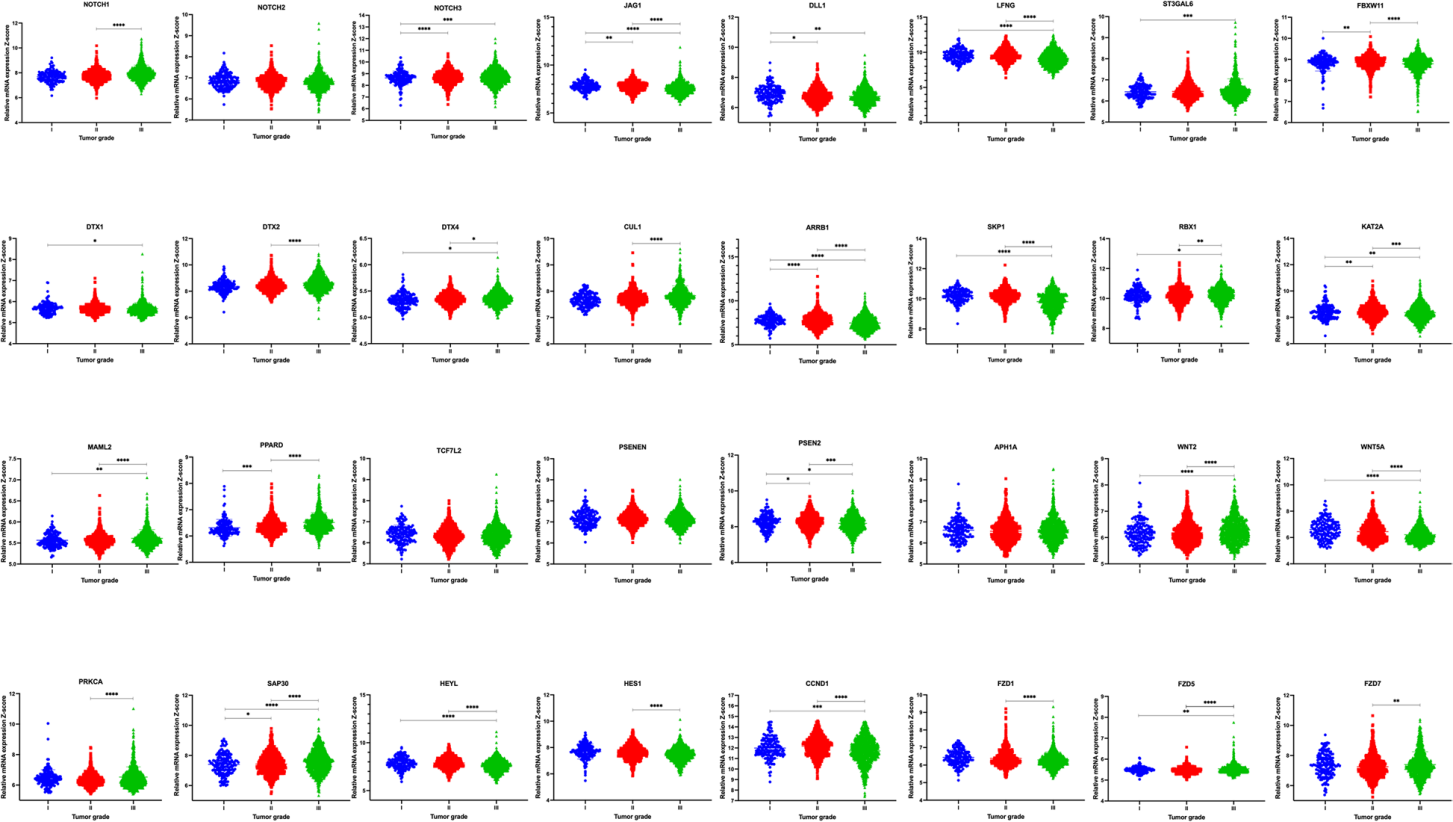
Relative expression of Notch signaling genes in different breast cancer tumor grades. Data were extracted from the METABRIC dataset and analyzed by one-way ANOVA followed by Tukey’s post hoc test. Tumor grade (I, *n* = 165; II, *n* = 740; III, *n* = 927). Statistically significant values of (*P*-value*: *P*-value < 0.05, **: *P*-value < 0.001, ***: 0.0001 < *P*-value = 0.0001, ****: *P*-value < 0.0001) were determined

**Fig. 4:**
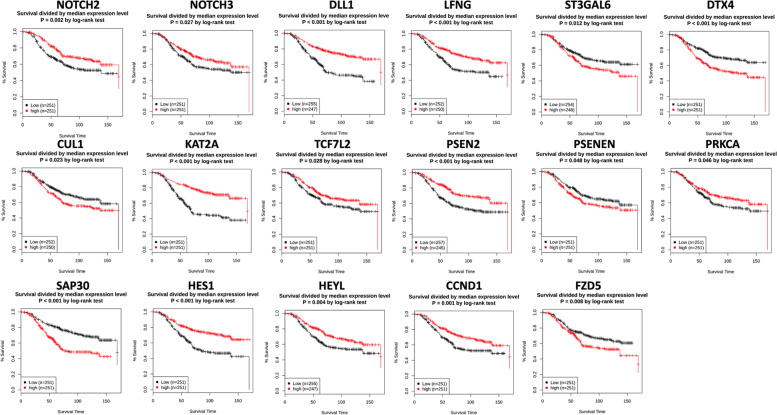
Notch signaling gene expression is associated with breast cancer survival. Kaplan–Meier overall survival (OS) curves for patients with breast cancer divided by the median value into low and high Notch-related gene mRNA expression (n = 251 per group) using the GENT2 platform http://gent2.appex.kr/gent2/

The expression of NOTCH-1, NOTCH-2, and NOTCH-3 was compared in each breast cancer molecular subtype. NOTCH-1 levels are significantly higher in the basal/claudin-low subtype than other subtypes (*P*-value < 0.0001). The up-regulation of NOTCH-2 has been seen in basal/claudin-low subtype compared to all other subtypes (*P*-value < 0.0001) except Luminal A. Similarly, NOTCH-3 was highly expressed in basal/claudin-low subtype relative to Luminal B subtype (*P*-value < 0.0001); however, its expression had no significant difference when compared to other subtypes and normal group. In contrast, there was significant alteration among other subtypes: HER2 vs. Luminal A (*P*-value = 0.0008) and Luminal B (*P*-value < 0.0001), Luminal A vs. Luminal B (*P*-value < 0.0001) and Luminal B vs. normal group (*P*-value < 0.0001), (Fig. 1a, Tables 1 and 2). In contrast with other NOTCH ligands, the NOTCH4 expression mRNA level was significantly lower in TNBC than in Luminal. Also, tumors with higher stages showed lower expression of NOTCH4 (stage III vs. Stage II; p-value: 0.0015), Supplementary Figure 1 a, f. Among Notch receptors, NOTCH-2 expression showed no significant change among different tumor grades and stages. However, NOTCH-1 and NOTCH-3 were positively correlated with the tumor grade and stage (Figs. 2 and 3, Tables 3 and 4). We also observed a significant negative correlation between NOTCH-1, NOTCH-2, and NOTCH-3 expression and their methylation, suggesting a potential epigenetic regulation of Notch receptors in breast cancer (Table 5). Survival analyses revealed a better survival for patients with higher expression of levels of NOTCH-2 (*P*-value = 0.002) and NOTCH-3 *(P*-value = 0.027), while NOTCH-1 had no significant effect on survival (Fig. 4).

**Table 1:** Notch signaling genes are differentially expressed across different breast cancer intrinsic subtypes

Genes	subtypes									
	Basal/Claudin-low Vs. HER2	Basal/Claudin-low Vs. Luminal A	Basal/Claudin-low Vs. Luminal B	Basal/Claudin-low Vs. Normal	HER2 Vs. Luminal A	HER2 Vs. Luminal B	HER2 Vs. Normal	Luminal A Vs. Luminal B	Luminal A Vs. Normal	Luminal B Vs. Normal
NOTCH-1	< 0.0001	< 0.0001	< 0.0001	< 0.0001	< 0.0001	< 0.0001	0.0239	< 0.0001	< 0.0001	< 0.0001
NOTCH-2	< 0.0001	0.0280	< 0.0001	0.0290	< 0.0001	0.8522	0.0410	0.0410	0.2445	0.0139
NOTCH-3	0.2749	0.0535	< 0.0001	0.9757	0.0008	< 0.0001	0.2773	< 0.0001	0.1330	< 0.0001
JAG1	< 0.0001	0.0208	< 0.0001	< 0.0001	< 0.0001	0.5840	< 0.0001	< 0.0001	< 0.0001	< 0.0001
DLL1	0.0008	0.7272	< 0.0001	< 0.0001	0.0002	0.0003	< 0.0001	< 0.0001	< 0.0001	< 0.0001
LFNG	< 0.0001	< 0.0001	< 0.0001	< 0.0001	0.4220	0.0726	0.0125	0.0897	0.0094	0.2392
ST3GAL6	< 0.0001	< 0.0001	< 0.0001	< 0.0001	0.0376	0.8781	0.0260	0.0046	0.4143	0.0116
FBXW11	0.0163	< 0.0001	< 0.0001	0.1405	< 0.0001	< 0.0001	0.0042	< 0.0001	0.0094	< 0.0001
DTX1	0.8746	0.0003	< 0.0001	0.3039	0.0076	< 0.0001	0.3279	< 0.0001	< 0.0001	< 0.0001
DTX2	< 0.0001	< 0.0001	< 0.0001	0.0772	< 0.0001	< 0.0001	< 0.0001	< 0.0001	0.0013	< 0.0001
DTX4	0.8560	< 0.0001	< 0.0001	0.5745	< 0.0001	< 0.0001	0.5069	0.2852	< 0.0001	0.0009
CUL1	< 0.0001	< 0.0001	< 0.0001	< 0.0001	< 0.0001	< 0.0001	< 0.0001	0.7459	0.0227	0.0375
ARRB1	< 0.0001	< 0.0001	< 0.0001	< 0.0001	< 0.0001	0.0015	< 0.0001	0.0176	0.7954	0.1000
SKP1	< 0.0001	< 0.0001	< 0.0001	< 0.0001	< 0.0001	< 0.0001	< 0.0001	0.0168	< 0.0001	< 0.0001
RBX1	< 0.0001	0.0794	0.0168	< 0.0001	< 0.0001	0.4207	0.2319	0.0002	< 0.0001	0.1287
KAT2A	0.9532	< 0.0001	< 0.0001	0.0070	< 0.0001	0.0001	0.0430	0.5435	0.4026	0.2728
MAML2	< 0.0001	< 0.0001	< 0.0001	0.0005	0.4603	0.0992	< 0.0001	0.0011	< 0.0001	< 0.0001
PPARD	0.3814	< 0.0001	< 0.0001	< 0.0001	< 0.0001	< 0.0001	< 0.0001	0.7865	0.1813	0.1579
TCF7L2	< 0.0001	< 0.0001	< 0.0001	0.1177	0.6095	< 0.0001	< 0.0001	< 0.0001	< 0.0001	< 0.0001
PSENEN	< 0.0001	< 0.0001	< 0.0001	0.2374	< 0.0001	0.3534	< 0.0001	0.0007	< 0.0001	< 0.0001
PSEN2	< 0.0001	< 0.0001	< 0.0001	0.0234	0.5739	< 0.0001	< 0.0001	< 0.0001	< 0.0001	0.0061
APH1A	0.3314	0.2413	0.0303	< 0.0001	0.0256	0.4093	< 0.0001	< 0.0001	< 0.0001	< 0.0001
PRKCα	< 0.0001	< 0.0001	< 0.0001	< 0.0001	< 0.0001	0.9256	< 0.0001	< 0.0001	< 0.0001	< 0.0001
SAP30	< 0.0001	0.6201	0.0001	< 0.0001	< 0.0001	0.0773	0.0030	0.0002	< 0.0001	0.0007
HEY1	0.0321	< 0.0001	0.4648	< 0.0001	< 0.0001	0.0816	0.0001	< 0.0001	0.4229	< 0.0001
HES1	< 0.0001	< 0.0001	< 0.0001	< 0.0001	0.0354	0.0262	0.0004	< 0.0001	0.0325	< 0.0001
CCND1	< 0.0001	< 0.0001	< 0.0001	< 0.0001	< 0.0001	< 0.0001	0.0177	< 0.0001	< 0.0001	< 0.0001
WNT2	0.8885	< 0.0001	< 0.0001	< 0.0001	< 0.0001	< 0.0001	< 0.0001	0.2228	0.0057	0.0024
WNT5A	0.2470	< 0.0001	0.0449	< 0.0001	< 0.0001	0.5831	0.0001	< 0.0001	0.0065	0.0005
FZD1	< 0.0001	0.2190	< 0.0001	0.2373	< 0.0001	0.0351	< 0.0001	< 0.0001	0.0269	< 0.0001
FZD5	0.0003	< 0.0001	< 0.0001	< 0.0001	< 0.0001	0.0028	0.0543	0.0056	0.0383	0.7824
FZD7	< 0.0001	< 0.0001	< 0.0001	0.4576	< 0.0001	0.0500	< 0.0001	< 0.0001	< 0.0001	< 0.0001

Representative statistics (*p*-values) are from RNA-Seq data for genes involved in Notch signaling from METABRIC by Pam50 gene expression subtype classification. Data were analyzed by one-way ANOVA followed by Tukey’s post hoc test. Statistically significant values of *p < 0.05, **p < 0.01, ***p < 0.001 and ****p < 0.0001 were determined

**Table 2:** Notch signaling genes are differentially expressed across different breast cancer intrinsic subtypes

Genes	subtypes									
	Basal/Claudin-low Vs. HER2	Basal/Claudin-low Vs. Luminal A	Basal/Claudin-low Vs. Luminal B	Basal/Claudin-low Vs. Normal	HER2 Vs. Luminal A	HER2 Vs. Luminal B	HER2 Vs. Normal	Luminal A Vs. Luminal B	Luminal A Vs. Normal	Luminal B Vs. Normal
NOTCH-1	0.5705	0.7185	0.8889	0.4662	0.1481	0.3185	-0.1043	0.1704	-0.2523	-0.4227
NOTCH-2	0.1703	0.05276	0.176	0.08958	-0.1176	0.00567	-0.08076	0.1233	0.03683	-0.08643
NOTCH-3	-0.06546	0.07726	0.3458	0.002168	0.1427	0.4112	0.06763	0.2685	-0.07509	-0.3436
JAG1	0.2552	-0.08598	0.2793	-0.2756	-0.3411	0.02411	-0.5307	0.3653	-0.1896	-0.5549
DLL1	0.1577	0.01206	0.3112	-0.2711	-0.1456	0.1535	-0.4288	0.2991	-0.2831	-0.5823
LFNG	-1.085	-1.028	-0.9328	-0.8226	0.057	0.152	0.2623	0.09504	0.2053	0.1103
ST3GAL6	0.4489	0.3943	0.4532	0.3689	-0.05451	0.004347	-0.07997	0.05886	-0.02546	-0.08432
FBXW11	0.09856	-0.1418	-0.2242	-0.06036	-0.2404	-0.3228	-0.1589	-0.08242	0.08147	0.1639
DTX1	0.004355	0.05787	0.1372	-0.02928	0.05351	0.1329	-0.03363	0.07934	-0.08715	-0.1665
DTX2	-0.241	0.2525	0.4197	0.1062	0.4935	0.6607	0.3471	0.1671	-0.1464	-0.3135
DTX4	-0.00222	0.05364	0.04568	0.007635	0.05585	0.0479	0.009852	-0.00796	-0.046	-0.03804
CUL1	0.1253	0.2535	0.2479	0.3081	0.1282	0.1226	0.1828	-0.00563	0.05461	0.06023
ARRB1	-0.2722	-0.6297	-0.5054	-0.6481	-0.3575	-0.2332	-0.3759	0.1243	-0.01845	-0.1427
SKP1	-0.297	-0.8474	-0.9083	-0.6829	-0.5504	-0.6113	-0.3859	-0.06089	0.1645	0.2254
RBX1	0.1089	0.142	-0.06428	0.1985	0.03308	-0.1732	0.0896	-0.2062	0.05653	0.2628
KAT2A	0.003187	-0.1908	-0.2121	-0.1482	-0.194	-0.2153	-0.1514	-0.02132	0.04258	0.0639
MAML2	0.19	0.1819	0.2092	0.0813	-0.00809	0.01912	-0.1087	0.02721	-0.1006	-0.1279
PPARD	-0.02952	0.2491	0.2538	0.2128	0.2786	0.2833	0.2423	0.004739	-0.03626	-0.041
TCF7L2	0.1925	0.177	0.4829	-0.07302	-0.01549	0.2904	-0.2655	0.3059	-0.25	-0.556
PSENEN	-0.2161	-0.1927	-0.3158	0.04409	0.02336	-0.09966	0.2602	-0.123	0.2368	0.3599
PSEN2	-0.2381	-0.3918	-0.4069	-0.09983	-0.1536	-0.1687	0.1383	-0.01507	0.2919	0.307
APH1A	-0.04979	0.04075	-0.08864	0.2834	0.09054	-0.03885	0.3332	-0.1294	0.2426	0.372
PRKCA	0.6862	0.6888	0.8771	0.419	0.002588	0.1909	-0.2672	0.1883	-0.2698	-0.4582
SAP30	0.2507	0.3426	0.02529	0.5152	0.09191	-0.2254	0.2645	-0.3173	0.1726	0.4899
HEY1	-0.1247	-0.4304	-0.0336	-0.3869	-0.3057	0.09106	-0.2623	0.3968	0.04341	-0.3533
HES1	-0.2678	-0.3541	-0.1644	-0.4587	-0.08628	0.1034	-0.1909	0.1896	-0.1046	-0.2942
CCND1	-0.5467	-1.177	-1.449	-0.8084	-0.6308	-0.9024	-0.2617	-0.2717	0.369	0.6407
WNT2	-0.00594	0.2019	0.1701	0.3056	0.2079	0.176	0.3116	-0.03185	0.1037	0.1356
WNT5A	-0.05577	-0.4741	-0.08401	-0.2964	-0.4183	-0.02823	-0.2406	0.3901	0.1777	-0.2123
FZD1	0.2265	0.03646	0.1508	-0.05784	-0.1901	-0.07575	-0.2844	0.1143	-0.09431	-0.2086
FZD5	0.07263	0.1354	0.1105	0.1065	0.06279	0.03789	0.03388	-0.02489	-0.02891	-0.00401
FZD7	0.9775	0.6828	1.074	0.06305	-0.2947	0.09693	-0.9144	0.3916	-0.6197	-1.011

Representative statistics (mean difference) between expression log intensity levels are from RNA-Seq data for genes involved in Notch signaling from METABRIC by Pam50 gene expression subtype classification

**Table 3:** Relative expression of Notch signaling gene in different breast cancer tumor stages

Genes			Stages				Genes			Stages			
	I vs. II	I vs. III	I vs. IV	II vs. III	II vs. IV	III vs. IV		I vs. II	I vs. III	I vs. IV	II vs. III	II vs. IV	III vs. IV
NOTCH-1	0.0143	0.0107	0.0417	0.0069	0.7524	0.7778	NOTCH-2	0.3502	0.5768	0.3490	0.2090	0.7943	0.4366
NOTCH-3	< 0.0001	< 0.0001	0.0066	0.0007	0.3834	0.6573	JAG1	0.0002	0.1228	0.2212	0.8496	0.1270	0.6334
DLL1	0.0095	0.1667	0.2689	0.0913	0.4634	0.5982	LFNG	< 0.0001	0.3275	0.0333	0.0001	0.0229	0.7558
ST3GAL6	0.2210	0.6782	0.8930	0.2689	0.8747	0.9107	FBXW11	0.5876	0.0989	0.7855	0.0433	0.2275	0.9528
DTX1	0.7220	0.3348	0.2996	0.1933	0.2182	0.2814	DTX2	0.3956	< 0.0001	0.0718	0.0002	0.6390	0.0080
DTX4	0.4932	0.0323	0.1619	0.2424	0.8120	0.1464	CUL1	0.0224	0.0188	0.4039	0.0006	0.1450	0.2370
ARRB1	< 0.0001	0.2355	0.5304	0.9458	0.9218	0.5142	SKP1	0.7262	0.0016	0.1474	0.0032	0.6744	0.2024
RBX1	0.8067	0.6752	0.0804	0.3725	0.7382	0.6261	KAT2A	0.6120	0.8310	0.0245	0.9514	0.2406	0.5996
MAML2	0.3685	0.7398	0.6783	0.4737	0.2906	0.7233	PPARD	0.8883	< 0.0001	0.9781	< 0.0001	0.6905	0.9056
TCF7L2	0.2857	0.4813	0.1489	0.8292	0.0542	0.8509	PSENEN	0.0176	0.5459	0.1281	0.3245	0.6504	0.4252
PSEN2	0.8642	0.3681	0.0179	0.8279	0.0279	0.7245	APH1A	0.0030	0.8784	0.0209	0.0489	0.1758	0.4034
PRKCα	0.0004	0.6957	0.7418	0.0812	0.5846	0.6706	SAP30	0.0094	0.1345	0.1695	0.0162	0.5657	0.7915
HEYL	0.0049	0.0604	0.0026	0.0566	0.0016	0.1162	HES1	0.0787	0.1622	0.0092	0.0148	0.9601	0.7395
CCND1	0.0025	0.0862	0.3752	0.8402	0.0152	0.0318	WNT2	0.1826	0.2662	0.1033	0.7807	0.7522	0.1974
WNT5A	0.2552	0.4074	0.5965	0.0062	0.2165	0.1957	FZD1	0.0108	0.0017	0.0155	0.1708	0.6474	0.9315
FZD5	0.6428	0.6570	0.4258	0.7177	0.0031	0.5106	FZD7	0.2082	0.0366	0.5839	0.2594	0.8440	0.7226

Representative statistics (*P*-values) between different tumor stages have been reported. Data were analyzed by one-way ANOVA followed by Tukey’s post hoc test. Statistically significant values of (*P*-value*: *P*-value < 0.05, **: *P*-value < 0.001, ***: 0.0001 < *P*-value = 0.0001, ****: *P*-value < 0.0001) were determined

**Table 4:** Relative expression of Notch signaling gene in different breast cancer tumor grades

Genes		Grades		Genes		Grades		Genes		Grades	
	I vs. II	I vs. III	II vs. III		I vs. II	I vs. III	II vs. III		I vs. II	I vs. III	II vs. III
NOTCH-1	0.3595	0.1562	< 0.0001	NOTCH-2	0.2223	0.0866	0.8627	NOTCH-3	< 0.0001	0.0010	0.0569
JAG1	0.0042	< 0.0001	< 0.0001	DLL1	0.0297	0.0060	0.7474	LFNG	0.0996	< 0.0001	< 0.0001
ST3GAL6	0.6187	0.5527	0.0002	FBXW11	0.0074	0.4546	< 0.0001	DTX1	0.1873	0.0427	0.7705
DTX2	0.7623	0.4963	< 0.0001	DTX4	0.0731	0.0210	0.0193	CUL1	0.6856	0.2127	< 0.0001
ARRB1	< 0.0001	< 0.0001	< 0.0001	SKP1	0.9002	< 0.0001	< 0.0001	RBX1	0.6319	0.0266	0.0094
KAT2A	0.0016	0.0025	0.0007	MAML2	0.2901	0.0076	< 0.0001	PPARD	0.0005	0.4582	< 0.0001
TCF7L2	0.2331	0.0683	0.1344	PSENEN	0.3173	0.9196	0.0917	PSEN2	0.0272	0.0432	0.0002
APH1A	0.1841	0.5830	0.0619	PRKCα	0.1473	0.1239	< 0.0001	SAP30	0.0215	< 0.0001	< 0.0001
HEYL	0.0758	< 0.0001	< 0.0001	HES1	0.2821	0.1110	< 0.0001	CCND1	0.2042	0.0005	< 0.0001
WNT2	0.0524	< 0.0001	< 0.0001	WNT5A	0.0713	< 0.0001	< 0.0001	FZD1	0.2537	0.1015	< 0.0001
FZD5	0.4319	0.0017	< 0.0001	FZD7	0.7096	0.4725	0.0049				

Representative statistics (*P*-values) between different tumor grades have been summarized. Data were analyzed by one-way ANOVA followed by Tukey’s post hoc test. Statistically significant values of (*P*-value*: *P*-value < 0.05, **: *P*-value < 0.001, ***: 0.0001 < *P*-value = 0.0001, ****: *P*-value < 0.0001) were determined

**Table 5:** Correlation between Notch signaling gene expression and their DNA methylation status in breast cancer

Gene name	Pearson r	*P*-value	Gene name	Pearson r	*P*-value
WNT5A	-0.13817	0.000103	ARRB1	-0.32126	0
WNT2	-0.11298	0.001522	CCND1	-0.50111	0
TCF7L2	-0.50965	0	CUL1	-0.24829	1.83E-12
ST3GAL6	-0.19776	2.31E-08	DLL1	-0.16074	6.1E-06
SKP1	-0.56178	0	DTX1	-0.37012	0
SAP30	-0.07777	0.029663	DTX2	-0.26772	2.35E-14
RBX1	-0.20721	4.63E-09	DTX4	-0.48782	0
PSENEN	-0.01787	0.617056	FBXW11	-0.21926	5.33E-10
PSEN2	-0.19644	2.87E-08	FZD1	-0.44727	0
PRKCA	-0.28897	0	FZD5	-0.24859	1.61E-12
NOTCH1	-0.5737	0	FZD7	-0.59981	0
NOTCH2	-0.19461	3.88E-08	HES1	-0.28982	0
NOTCH3	-0.17568	7.31E-07	HEYL	-0.06506	0.068493
PPARD	-0.42873	0	DTX2	-0.34271	0
MAML2	-0.42976	0	KAT2A	-0.29738	0
APH1A	-0.0384	0.282519	LFNG	-0.48564	0

TCGA breast invasive carcinoma DNA methylation (Illumina Infinium HumanMethylation450) and gene expression microarray datasets were analyzed. Representative statistics demonstrated correlation between Notch signaling gene expression and their DNA methylation status. Gene expressions were not found to be correlated with DNA methylation. Gene expressions were found to be negatively correlated with DNA methylation for most Notch pathway effectors. The Pearson correlation coefficients **(r)** and the relative ***p*-values** are shown. The association between genes was measured using the Pearson correlation coefficient (r) and respective computed P-value

### High expression of Notch Ligands is associated with the basal/claudin-low subtypes of breast cancer

It has been reported that there is a positive association between the high expression of Jagged1 (JAG1) and the metastasis of breast cancer cells by initiating epithelial to mesenchymal transition EMT [33]. The high level of JAG1 was also correlated with higher tumor stage and grade, and poor prognosis and survival rate in lymph node-negative breast cancer [23, 34]. The undetectable expression of DLL1 in normal breast tissues and its moderate to high expression in breast cancer [35]. Accordingly, our data showed that JAG1 and DLL1 expression levels were significantly elevated in metastatic subtype, basal/claudin-low (*P*-value = 0.0008 up to 0.0001) compared to HER2, Luminal B subtypes, and normal group. Jag1 was highly expressed (*P*-value = 0.02), in Basal/Claudin low compared to Luminal A, while DLL1 had no significant difference. (Fig. 1a, Tables 1 and 2). DDL3 has the highest expression in the Basal/claudin-low subtype compared to all other subtypes (*p*-value < 0.0001). Similarly, Jag2 expression was higher in the Basal/claudin-low subtype compared to the Luminal A and B subtypes (*P* value: 0.0018, *P* value < 0.0001, respectively). There was no significant differential expression of DLL3 and JAG2 between different tumor stages; however, tumors with higher grades showed significantly higher expression of DLL3 (grade III vs. grades I, II; *P* value < 0.0001). Also, grade III tumors showed higher JAG2 expression than grade II tumors (P value: 0.0387), Supplementary Figure 1. Both JAG1 (*P*-value = 0.0002) and DLL1 (*P*-value = 0.009) showed higher expression in stage I than stage II of breast cancer. JAG1 was also highly expressed in grade I of breast cancer compared to other grades, and in grade, I compared to grade II, while DLL1 expression was higher in grade II and III than grade I (Figs. 2 and 3, Tables 3 and 4). Interestingly, we found a significant negative correlation between the expression of JAG1 and DLL1 and the DNA methylation level (Table 5). Also, increased DLL1 expression was correlated with better survival in breast cancer patients (*P*-value < 0.001) JAG1 expression was not associated with patient survival (Fig. 4).

### Notch signaling modifier, LFNG is highly expressed in the HER2 subtype, while ST3GAL6 is highly expressed in the basal/claudin-low subtype

Sialyltransferases (STs) are a family of enzymes that act specifically to conjugate sialic acids to sugar molecules in glycans [36]. ST3GAL6 (β-galactose α-2, 3-sialyltransferase) modifies ligand synthesis, and its high level was associated with breast cancer metastasis [37]. Lunatic Fringe (Lfng) is a gene that encodes a β3N-acetylglucosaminyl-transferase, which regulates ligand-mediated activation of the Notch via Delta-like ligands DLL1 and DLL4, and inhibits Notch activity through Serrate/Jagged ligands JAG1 and JAG2 [38]. The deletion of LFNG is responsible for the altered differentiation pattern of luminal cells, the proliferation of basal cells, EMT, and increased population of stem-like cells; it was also correlated with poor survival of breast cancer patients [39]. Accordingly, our results showed LFNG expression was lower in basal/claudin-low breast cancer compared to other subtypes and normal group (*P*-value < 0.0001) while ST3GAL6 has the highest expression in basal/claudin-low breast cancer (*P*-value < 0.0001) (Fig. 1a, Tables 1 and 2). Also, LFNG expression was higher in stage II of breast cancer compared to other stages (*P*-value < 0.0001 up to 0.0229), in stage I compared to stage IV (*P*-value = 0.0333), and in grade III compared to other grades. ST3GAL6 expression was significantly higher in grade III than II of breast cancer (*P*-value = 0.0002); however, there was no significant change between different stages of breast cancer (Figs. 2 and 3, Tables 3 and 4). Interestingly, LFNG and ST3GAL6 expression were negatively associated with their DNA methylation status (Table 5). Furthermore, high expression of LFNG (*P*-value < 0.001) and lower expression of ST3GAL6 (*P*-value = 0.012) was significantly related to better survival in breast cancer (Fig. 4).

### FBXW11 and DTX family 1, 2, and 4, E3 ubiquitin ligases are significantly downregulated in HER2 and Luminal B subtypes, respectively

FBXW11 is a ubiquitin–proteasome system (UPS) that plays a key role in regulating various signaling pathways by post-translational modifications regulating cancer cell tumorigenesis [40]. DTX, E3 ubiquitin ligase Deltex also initiates the activation of the Notch signaling pathway in an independent-ligands manner [41]. We showed that FBXW11 was downregulated in HER2 and basal/claudin-low compared to the other subtypes (Tables 1 and 2). DTX family showed a significantly lower expression in Luminal B compared to all other subtypes (*P*-value < 0.0001) (except for the DTX4 expression in Luminal B compared to Luminal A (Fig. 1a, Tables 1 and 2). Nevertheless, significant associations with survival were observed in breast cancer patients for lower expression of DTX4 (*P*-value < 0.001) (Fig. 4). Interestingly, FBXW11 and all the studied DTX family gene expressions showed a negative correlation with their methylation level (Table 5). Moreover, FBXW11 and DTXs expression were correlated with tumor stage and grades, which has been summarized (Fig. 2, 3, Tables 3 and 4).

### Expression levels of the other members of E3 ubiquitin ligases (CUL1, ARRB1, and SKP1) are associated with the basal/claudin-low subtype

ARRB1 is a member of the β-arrestin protein family responsible for deactivation and degradation of receptors coupled with G protein [42]. ARRB1 initiates the degradation of NOTCH1 in *Drosophila* and mice by regulating the interaction between NOTCH1 and E3 ubiquitin ligases DTX1 [43]. SKP1-CUL1-F-box is also an E3 ligases family and regulates the Notch signaling pathway [44]. SKP1-CUL1 is associated with proteins consisting of the RING protein Ring-box 1 (RBX1) to regulate the β-catenin ubiquitination and degradation of their targets [45]. Although a significantly high expression of CUL1 was observed in the basal/claudin-low subtype (*P*-value < 0.0001), the expression of ARRB1 and SKP1 was markedly lower in basal/claudin-low relative to other subtypes and the normal group (*P*-value < 0.0001). We showed that the RBX1 expression is higher in Luminal than all other subtypes (Fig. 1a, b, Tables 1 and 2). In addition, their expression was correlated with the disease grade and stage (Fig. 2, 3, Tables 3 and 4). We also showed that CUL1, ARRB1, SKP1, and RBX1 gene expression was negatively correlated with DNA methylation (Table 5). Among these factors, it was shown that lower expression of CUL1 had a positive effect on survival rate (*P*-value = 0.023) (Fig. 4).

### KAT2A, MAML2, PPARD, and TCF7L2 are highly expressed in basal/claudin-low subtype

KAT2A is a member of the histone acetyltransferases (HATs) family, which function as a transcription coactivator [46]. MAML2 (mastermind‐like transcriptional coactivator 2) is also a transcription coactivator that regulates the Notch signaling pathway and can function as an oncogene in breast cancer. The upregulation of PPARD (peroxisome proliferator-activated receptor–δ) as a nuclear transcriptional receptor has been reported in various cancers by regulating metastasis [47]. TCF7L2 is a transcription factor and acts as an effector of the WNT pathway, a counterpart of the Notch signaling pathway [48]. KAT2A and PPARD were highly expressed in the basal/claudin-low subtype compared to other subtypes (*P*-value = 0.007 up to 0.0001) except for HER2, which had no significant change. Similarly, high expression levels of MAML2 and TCF7L2 were seen in basal/claudin-low compared to all other subtypes and the normal group (*P*-value < 0.0001) (Fig. 1b, Tables 1 and 2). Interestingly, gene expression levels of these regulators were negatively correlated with their DNA methylation status (Table 5). Furthermore, KAT2A and PPARD showed different expression levels based on the tumor stage, while TCF7L2 expression showed no significant change between different tumor grades (Fig. 2, 3, Tables 3 and 4). We also observed that high expression levels of KAT2A (*P*-value < 0.001) and TCF7L2 (*P*-value = 0.0228) were correlated with better survival rates in patients (Fig. 4).

### The methylation pattern of Gamma-Secretase Complex PSENEN and APH1A were not significantly altered

Presenilin enhancer protein 2 (PSENEN) and anterior pharynx-defective 1 (APH1-) are members of the gamma-secretase complex [49]. The γ-secretase activates the Notch signaling pathway through the cleavage of Notch receptors, type I transmembrane proteins, and amyloid precursor protein [50]. High expression of PSENEN was associated with the Luminal B subtype (Fig. 1b). PSEN2 has the highest expression level in the Luminal B subtype, compared to all other subtypes. Expression of APH1A was similar between basal/claudin-low and HER2 subtype and higher compared to normal group (*P*-value < 0.0001) (Fig. 1b, Tables 1 and 2). PSENEN and APH1A expression were correlated with tumor stage but not the tumor grade (Figs. 2 and 3, Tables 3 and 4). PSEN2 gene expression level showed a significant negative correlation with its DNA methylation (Table 5). Although the correlation between the gene expression level of PSENEN and APH1A and their DNA methylation was negative, however, it was not meaningful (Table 5). Nonetheless, lower PSENEN (*P*-value = 0.048) and higher PSEN2 (*P*-value < 0.001) expressions were significantly associated with better survival in breast cancer (Fig. 4).

### PRKCα and SAP30 were highly expressed in basal and luminal A subtypes, respectively

PKC (Protein kinase C) is a serine/ threonine kinases family that contributes to cellular differentiation, proliferation, and survival [51]. SAP30 is a nuclear protein that regulates transcriptional repression through its histone deacetylase activity and recruiting nuclear hormone receptors and homeodomain proteins [52]. PRKCα was highly expressed in basal/claudin-low subtype compared to other subtypes (*P*-value < 0.0001). SAP30 was highly expressed in the basal/claudin-low compared to all subtypes (Fig. 1b, Tables 1 and 2). Expression levels of both PRKCα were negatively correlated with DNA methylation status (Table 5). Moreover, their expression was associated with the tumor stage and grade (Figs. 2 and 3, Tables 3 and 4). Also, PRKCα's high expression (*P*-value = 0.046) and SAP30 lower expression (*P*-value < 0.001) were associated with a higher survival rate in breast cancer (Fig. 4).

### High expression of target genes of the Notch signaling pathway (HEYL, HES1, and CCND1) are significantly associated with better survival rates

HEYL, HES1, and CCND1 are considered primary targets of Notch signaling [53–55]. HEYL (hairy/enhancer-of-split related with YRPW motif) is a known transcriptional target of the Notch pathway, and its overexpression has been shown in breast cancer [56, 57]. Finally, Cyclin D1 (CCND1) is a direct target of Notch signaling and contributes to breast cancer cell cycle progression and proliferation [58]. While HES1 and CCND1 were highly expressed in the Luminal B subtype (*P*-value = 0.0262 up to 0.0001), high expression of HEYL was associated with basal/claudin-low subtype (*P*-value < 0.0001) with no significant change compared to Luminal B (Fig. 1c, Tables 1 and 2). Among these three factors, HES1 and CCND1 gene expression levels negatively correlated with DNA methylation (Table 5). Gene expression patterns of these factors in different breast cancer subtypes, stages, and grades have been summarized (Fig. 2, 3, Tables 3 and 4). Furthermore, increased expression of these factors was associated with better survival in breast cancer patients (Fig. 4I).

### WNT pathway receptors are associated with basal/claudin-low subtype

Interaction between Notch and WNT pathways has been shown previously [59]. Frizzled family receptors play an essential role in the activation of the WNT pathway by binding to the low-density lipoprotein receptor-related protein (LRP) [60]. We showed a significantly high expression level WNT2, and a lower expression of WNT5A in basal/claudin-low compared to other subtypes is significant (*P*-value = 0.0449 up to 0.0001) (Fig. 1c, Tables 1 and 2). The high expression of FZD1 was seen in basal/claudin-low relative to HER2 and Luminal B (*P*-value < 0.0001). Also, FZD5 has a significantly high expression in basal/claudin-low compared to other subtypes (*P*-value < 0.0001), however, in the case of FZD7, the higher expression was significant in both basal/claudin-low and normal groups relative to other subtypes (*P*-value < 0.0001) (Fig. 1, Tables 1 and 2). There was also a negative correlation between DNA methylation levels of WNT2, WNT5A, FZD1, and FZD7 and their gene expression (Table 5). Furthermore, these genes were differentially expressed based on the tumor stage and grade (Figs. 2 and 3, Tables 3 and 4). Moreover, the lower expression of FDZ5 had a positive effect on the survival rate in patients (*P*-value = 0.008) (Fig. 4).

### Dysregulation of Notch pathway gene expression in breast cancer tissues compared to normal samples

**Fig. 5:**
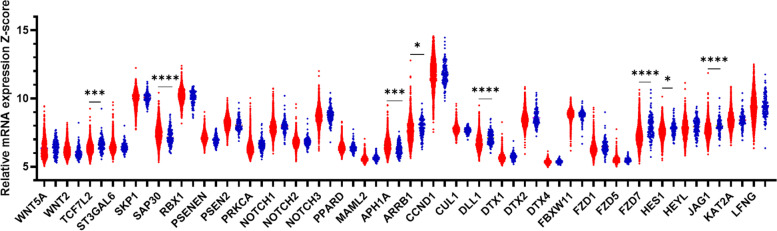
Notch signaling pathway gene expression pattern in breast cancer and normal tissues. RNA-Seq mRNA expression data for breast cancer (red color) and normal (blue in color) subtypes in METABRIC cohort. Normal (Normal-like) samples (n = 148) and cancer tissues (all subtypes), (n = 1826) of primary breast tumors have been used. Data were analyzed by an unpaired t-test between cancer and normal group for each single gene. Statistically significant values of *p < 0.05, **p < 0.01, ***p < 0.001 and ****p < 0.0001 were determined

Here we assessed differential expression of Notch pathway genes at the mRNA level in normal and breast cancer tissues. mRNA expression of SAP30 and APH1A were significantly up regulated in breast cancer tumors compared to control tissues from healthy individuals (*p* < 0.0001 and *p* = 0.0001, respectively) (Fig. 5). On the other hand, TCF7L2, ARRB1, DLL1, FZD7, HES1, and JAG1 transcript levels were significantly lower in breast cancer tissues than in normal samples (*p* < 0.0009, *p* = 0.0131, *p* =  < 0.0001, *p* =  < 0.0001, *p* = 0.0138, and *p* < 0.0001, respectively) (Fig. 5). We do not expect to observe deregulated or upregulated mRNA levels of all Notch genes in breast cancer because the biological functions of some genes, particularly those coding for kinases, depend on the phosphorylation of their proteins. Therefore, to clarify the exact association of such genes with cancer, protein phosphorylation detecting strategies such as immunohistochemistry and Western blotting staining are essential to provide additional support.

### Notch signaling pathway positively correlated to many tumor-evasion, metastasis, and angiogenesis markers in breast cancer

**Fig. 6:**
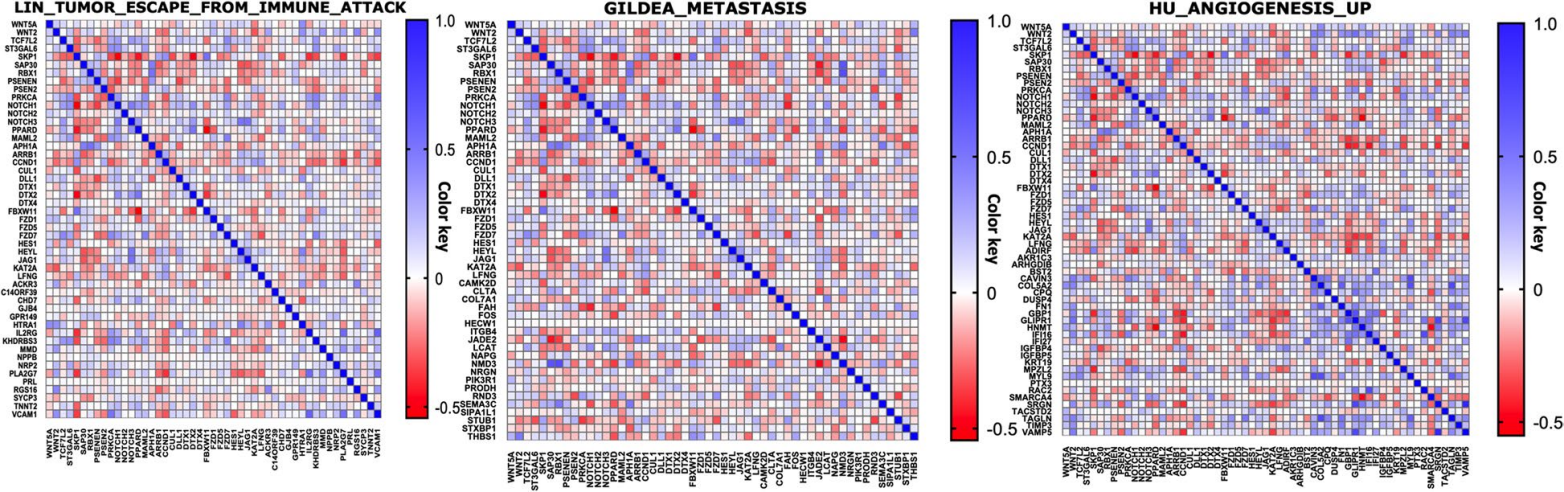
Notch signaling pathway is positively correlated with tumor-evasion, metastasis and angiogenesis markers. Co-expression (mRNA) correlation analysis between Notch pathway elements and tumor-evasion, metastasis and angiogenesis markers using **a**, LIN_TUMOR_ESCAPE_FROM_IMMUNE_ATTACK **b**, GILDEA_METASTASIS, and **(c)**, HU_ANGIOGENESIS_UP gene sets, respectively (https://www.gsea-msigdb.org/gsea/msigdb). Correlation heatmap (Pearson r) of the transcriptomes from METABRIC breast cancer project samples (n = 1986). The red color refers to negative correlation, and the blue color indicates positive correlation. Immune-evasion-related genes (genes up-regulated in highly immune-resistant cancer cells) comprised 20 genes. The metastasis-related genes comprised 29 genes. The angiogenesis-related genes contained 21 genes. The mRNA expression data were extracted from METABRIC. Pearson's correlation analysis was used for the analysis of co-expression between genes

Notch signaling has been shown to be involved in regulating many components of the tumor microenvironment [61]. Increasing evidence has highlighted the vital role of Notch in breast cancer metastasis [31]. During angiogenesis, Notch ligands and Notch-associated proteases cooperate with vascular endothelial growth factor (VEGF) to establish neovasculatures to promote breast cancer metastasis [31]. Moreover, Notch signaling pathway also crosstalk with other pathways including Hedgehog, TGF-β and AKT signaling pathways [62]. Based on the analysis of the clinical data from the METABRIC study, we showed that the Notch signaling pathway has a significant positive correlation with many tumor-evasion, metastasis, and angiogenesis markers (Fig. 6 a, b, and c). In addition, Notch-regulating elements showed co-expression correlation with multiple factors playing role in Hedgehog, TGF-β and AKT signaling pathways, Supplementary Figure 2. A, b, and c. This result might support the previously known Notch signaling tumorigenic effects through regulating tumor-evasion, cancer stemness, metastasis, angiogenesis, and crosstalk with other pathways in breast cancer.

### Multiple MicroRNAs and long non-coding RNAs might be involve in regulating the Notch pathway

**Fig. 7:**
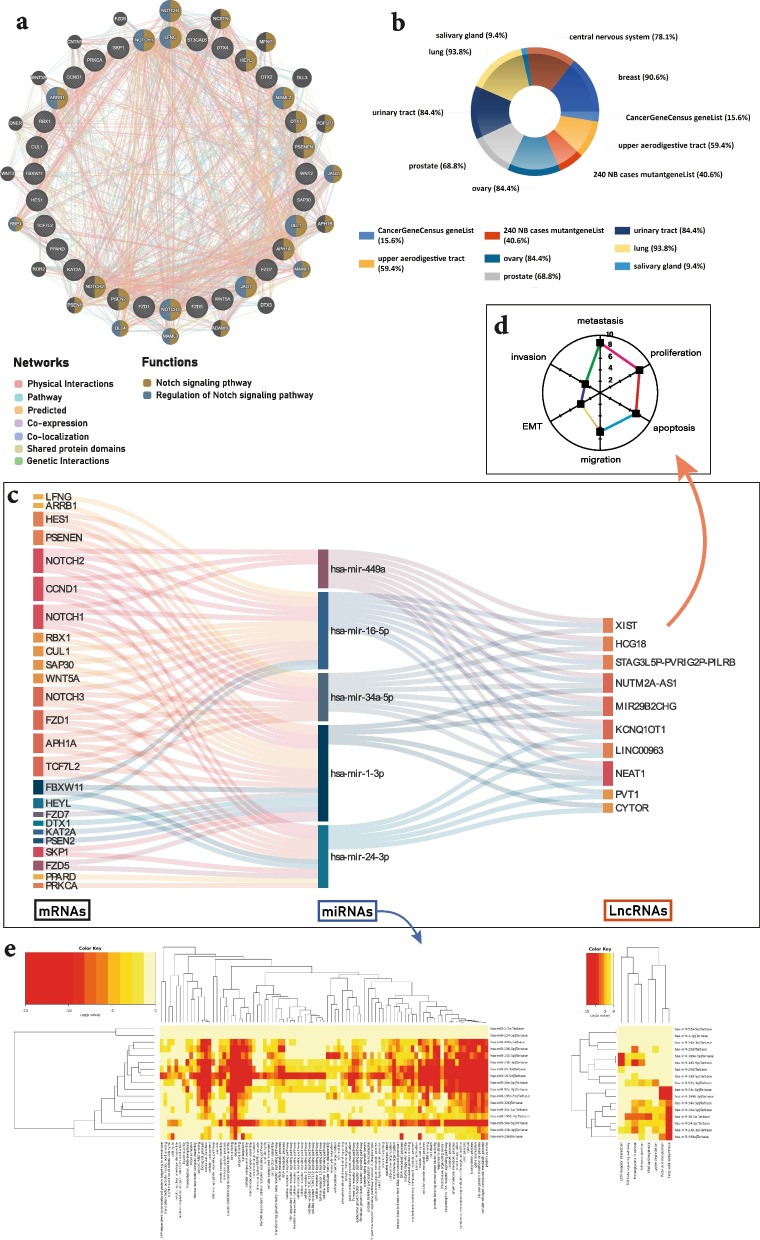
Non-coding RNA interaction with Notch signaling pathway. **a** The protein–protein interaction (PPI) interaction networks for the studied genes using the GeneMANIA database. Nodes represent genes, and the node color represents the possible function of the respective gene. **b** Using FunRich v.1.3.4 performed COSMIC, which shows breast cancer is common cancer between 90.6% of studied genes. **c** Sankey diagram for the mRNAs-miRNAs-LncRNAs network. Each rectangle represents a gene, and the size of the rectangle is based on the degree of the connection for each gene. **d** The common Cancer-Hallmarks between LncRNA targets of miRNAs in this study were performed by the LncSEA database. **e** GO-heatmap and Pathway-heatmap of miRNA targets of studied mRNAs are generated from DIANA-miRPath v3.0

Recent studies suggest that MicroRNAs (miRNAs) and long non-coding RNAs (lncRNAs) are dysregulated in many cancers by regulating different tumorigenic pathways [29, 30, 63]. They are also involved in regulating the Notch pathway through modulating the expression of receptors or ligands at different steps, including transcriptional or posttranscriptional levels [64]. Therefore, developing functional computational models and networks to predict the potential Notch-miRNA/lncRNA association may benefit not only understanding the Notch regulatory mechanisms at the non-coding RNA level, but also the detection of breast cancer biomarkers for disease diagnosis, treatment, and prognosis. Accordingly, the 32 candidate mRNAs in this study were subjected to further analysis to understand their roles in breast cancer by interacting with predicted miRNAs and LncRNAs as possible targets. Using bioinformatic analyses five miRNAs (hsa-miR-1-3p, hsa-miR-16-5p, hsa-miR-24-3p, hsa-miR-34a-5p, hsa-miR-449a) and 10 LncRNAs (NEAT1, KCNQ1OT1, MIR29B2CHG, NUTM2A-AS1, XIST, HCG18, LINC00963, STAG3L5P-PVRIG2P-PILRB, CYTOR, PVT1) were found (Fig. 7c, d, and e). The protein–protein interaction PPI networks constructed by GENEMANIA showed the pathways, genetic interaction, shared protein domains, co-expression, co-localization networks, as well as predicted interactions between studied mRNAs (shown by different colors in Fig. 7a). The COSMIC enrichment analysis showed that 90.6% of the input genes might be involved in breast cancer (Fig. 7b).

## Discussion

Targeted therapies mean using the specific overexpressed proteins on the cell surface that are the main reasons for the unlimited growth of the cells to treat or decrease disease progression. Targeted therapies for breast cancer consist of using drugs in order to block the function of molecules related to survival and cell proliferation so that the growth of breast cancer is terminated [65]. Estrogen and estrogen receptors are important factors in the progression of breast cancer. Positive estrogen receptor (ER +) is expressed in about 75% of patients so it is the main cause of breast cancers [66, 67]. Therefore, direct target of estrogen signaling pathway and estrogen receptors, such as selective estrogen receptor modulators and aromatase enzyme, has been used as a therapeutic approach for patients by inhibiting tumor growth [68]. Another common receptor, HER2, is overexpressed in about 25% of patients, which is responsible for a more aggressive type of tumor and is related to poor prognosis in breast cancer patients [69]. The drugs used to treat this kind of breast cancer have been demonstrated to be susceptible to the development of resistance mechanisms, resulting in considerable side effects for the patients [70]. The lack of estrogen, progesterone, and HER2 is known as triple negative breast cancer (TNBC). TNBC is correlated with poor prognosis, and no treatment has been introduced for this subtype yet [71]. The lack of any specific and effective target in few subtypes and the rapid spread of resistance toward existed targeted therapies have negatively influenced efficacy of treatment, therefore it is of great importance to develop and determine novel pathway blockades and therapeutic targets specific to each subtypes in order to raise the probability of successful treatment [65]. The identification of new pathways and target genes in breast cancer and the application of these separately and in combination as targeted therapies may have improved the effectiveness of treatment and patients’ outcomes by overcoming the resistance mechanisms. For example, in TNBCs, the lack of fully understood proliferative pathways seems to be the most important limitation of patients’ treatment [72].

Notch signaling is a highly conserved pathway that plays crucial roles during mammary gland development and is the primary regulator of cell differentiation and fate determination [73–75]. The oncogenic role of this pathway has been shown in breast cancer that induces the normal epithelial cell transformation into malignant breast cancer cells [76]. Dysregulation of the Notch pathway has been reported in different breast cancer subtypes to modulate tumor progression through various mechanisms such as resistance to drug treatment [16, 77, 78]. Therefore, our study aims to find the unique expression pattern of each Notch pathway factor in an individual tumor subtype that could be specific promising therapeutic targets.

NOTCH-1, NOTCH-2, and NOTCH-3 expression analysis showed that the basal/claudin-low subtype has the highest levels of NOTCH-1 compared to other subtypes. Additionally, there was a negative correlation between NOTCH-1 expression and its DNA methylation, suggesting a critical role of epigenetic regulation, especially in the basal/claudin-low subtype. One potential mechanism is the activation of NF-kB signaling by NOTCH-1, resulting in invasive growth and migration of breast cancer cells [79]. NOTCH-2 was also upregulated in the basal/claudin-low subtype. In metastatic TNBC, NOTCH-2 amplification has been linked to a poor prognosis [80]. In MDA-MB-231 cells, however, NOTCH-2 has been shown to have antitumor effects [80]. NOTCH-3 was highly expressed in the basal/claudin-low subtype, but its elevated expression was only significant relative to the Luminal B subtype and not the other subtypes. Previously, NOTCH-3 expression has been connected to breast cancer metastasis by inducing breast cancer cell invasiveness and stemness [81]. NOTCH-3 was differently expressed in different breast cancer stages and grades; it was highly expressed in stage and grade III compared to lower stage and grades. The up-regulation of both NOTCH-2 and NOTCH-3 were correlated with longer survival rates in breast cancer patients.

Members of the Notch family can play as both tumor suppressors or oncogene based on the context of the disease [82]. JAG1 is a major component of the Notch pathway and has been linked to normal biological processes, including tissue homeostasis, adult stem cell control, and oncogenic functions, including carcinogenesis, vascularization, and tumor growth [83, 84]. JAG1 has shown to be associated with poor overall survival in women with advanced breast cancer and disease recurrence [34, 85]. Similarly, YAP1 has previously been linked to oncogenic phenotypes in breast cancer cells by acting at the upstream level of the Notch pathway and upregulating JAG1 expression [86]. On the other hand, DLL1 has also been shown to boost tumor cancer stem cells in renal cell carcinoma and maintain stem cell phenotype in glioblastoma [87, 88]. Recent studies have shown that Notch ligand DLL1 is highly expressed in ER + luminal breast cancer and elevated levels of DLL1 mediate tumorigenesis and metastasis [89]. Our data showed that JAG1 and DLL1 were highly expressed in the basal/claudin-low subtype compared to HER2, Luminal B, and normal groups. Our study demonstrated that JAG1 and DLL1 were shown to be elevated in stage I compared to stage II for both genes. Increased DLL1 expression was correlated with better survival in breast cancer. We also found a significant negative correlation between JAG1 expression and its DNA methylation level. ST3GAL6, a glycotransferase and the glycosyltransferase Lunatic Fringe (LFNG), also known as *O*-fucosylpeptide 3-beta-*N*-acetyl-glucosaminyltransferase are involved in Notch signaling regulation [90, 91]. Accordingly, we found a high expression level of ST3GAL6 in basal/claudin-low subtypes, while LFNG is highly expressed in HER2 subtypes. The expression of LFNG was higher in stage II of breast cancer compared to the other stages and in grade III compared to the other grades. In the case of ST3GAL6, its expression was significantly higher in grade III than II of the tumors. Higher expression of LFNG was correlated with better survival in breast cancer, while ST3GAL6 expression was correlated with poor survival. High expression of ST3GAL6 has been found in different cancers, including breast, multiple myeloma, and hepatocellular carcinoma, whereas normal tissues showed lower expression levels [37, 92]. Xu et al. demonstrated that LFNG suppresses Jagged/Notch signaling in vivo, and it was consistently expressed at a low level in basal-like tumors, and deletion of LFNG in mouse mammary gland enhances the accumulation of activated Notch intracellular domain polypeptides resulting in an increase in cell proliferation and inducing the basal phenotypes [93]. The F-box protein β-TrCP1 (FBXW1A) triggers the ubiquitin-dependent proteolysis of the proteins to regulate DNA methylation patterns during DNA replication and tumor development [94]. FBXW1A expression was found to enhance cell transformation by activating NF-kB-dependent survival pathways. Higher FBXW11 expression is a factor that promotes cell transformation, and it has been shown that in mammary tissues of transgenic mice with negative FBXW11 expression, the development of breast tumors is reduced [95]. Deltex (DTX) family members control the transcription, and they exhibit ubiquitin-protein isopeptide ligase activity plying regulatory role in neurogenesis and Notch signaling pathway. DTX3 acts as a driver of proliferation in luminal breast cancer and its amplification was linked to a poor outcome in patients [93]. Our results indicated that FBXW11 was significantly downregulated in HER2 while the DTX family (1, 2, and 4, E3 ubiquitin ligase) was downregulated in the luminal B subtype.

Compared to benign neoplasia and normal breast samples, breast cancer samples had high expression levels and/or amplification of KAT2A [96]. MAML2 is the Notch activator through epigenetic regulation [97]. Through decreasing oxidative stress and increasing survival signaling responses, PPARD supports breast cancer cells to thrive under severe microenvironmental circumstances [98]. KAT2A and PPARD showed different expressions based on tumor stages and grades with favorable survival rates in patients. TCF7L2 has been detected on the promoter of the cyclin D1 gene in human breast cancer cells, indicating that it increases cyclin D1 expression. In 20% of human breast malignancies, cyclin D1 gene is amplified cyclin D1 protein is produced at a high level in 50% of human breast tumors [99]. We showed that CCND1 has the highest expression in luminal and the lowest in basal/claudin-low subtypes. Our finding suggests that KAT2A (histone acetyltransferase), MAML2 (recruiter of transcriptional coactivators), PPARD, and TCF7L2 (transcription factor) were highly expressed in the basal/claudin-low subtype. KAT2A and PPARD showed different expressions between tumor stage and grades. High expression of KAT2A and TCF7L2 (in all the subtypes) were correlated with better survival rates in patients.

High expression of Cul1 is linked to the development of breast cancer by promoting proliferation, migration, and invasion of breast cancer cells and is associated with a poor prognosis [100]. Studies have shown that Arrestin beta 1 (ARRB1) is particularly downregulated in TNBC patients. ARRB1 high expression inhibited TNBC cell proliferation and migration and is negatively correlated with breast cancer histological grade and favorably associated with TNBC patient survival, suggesting that ARRB1 has a tumor-suppressive function in breast cancer [101]. Skp1 is a part of the Skp1-Cullin1-F-box protein (SCF) E3 ligase complex, which has recently been involved in carcinogenesis and drug resistance through degradation of their downstream substrates [102]. In addition, RBX1 as a part of ROC1/RBX1 E3 ubiquitin ligase silencing inhibited tumor cell growth via sequential induction of G2/M arrest, apoptosis, and induction of senescence [103]. Although the basal/claudin-low subtype had a significantly high expression of CUL1 between all the subtypes, ARRB1 and SKP1 had the lowest expression levels in the basal/claudin-low subtype compared to all other subtypes. RBX1 expression was high in the Luminal B subtype.

Intramembrane-cleaving protease γ-Secretase is involved in Notch signaling coded by several genes, including PSENEN, PSEN2, and APH1A [104]. The activation of the Notch receptor is regulated by a sequence of proteolytic cleavages involving this complex [50]. Accordingly, inhibitors of γ-Secretase might be a therapeutic option by suppressing the Notch pathway. It has also been demonstrated that expression of PSENEN reduced in the PR-positive group (progesterone receptor-positive group) compared with the PR-negative group [105]. Studies indicated that many individuals diagnosed with breast cancer have PSEN2 R62H and PSEN2 R71W variations, and it was shown that these PSEN2 polymorphisms might predispose to cancer formation. Previous studies have shown that the PSEN2 N141I variation increases cancer risk by blocking NOTCH-mediated apoptosis; however, there is no evidence in the literature linking cancer risk to the PSEN2 R62H and R71W variants [106]. APH1A expression was the same in basal/claudin-low and HER2 subtypes and was more remarkable than the normal group. Low APH1A expression has been linked to a higher risk of breast cancer-specific death. Tumors expressing low amounts of APH1A had a high histological tumor grade, according to the findings [107]. PSENEN and PSEN2 high expression were associated with the Luminal B subtype. Lower PSENEN and higher PSEN2 expression are significantly associated with better survival in breast cancer. Whereas, in patients with breast cancer, it has been demonstrated that there is a statistically significant association between initial tumor expression of PSENEN and recurrence-free survival (RFS). Those with low levels of PSENEN in their primary tumors had a median RFS of 55 months, whereas patients with high levels of PSENEN had a median RFS of 45.93 months [108].

MCF-7 breast cancer cells transfected with protein kinase C-alpha (PKC-α) showed a higher proliferative rate, anchorage-independent growth, and increased tumorigenicity in vivo [109]. SAP30 is part of a multi-complex protein called SIN3A. Breast cancer cells require SIN3A for survival and development. The LSD1/SIN3A/HDAC complex has been shown to regulate breast cancer cells' survival and carcinogenic potential and is required for epithelial homeostasis and chemotherapeutic sensitivity [110]. Our data showed that PRKCα was highly expressed in the basal/claudin-low subtype when compared to other subtypes. Similarly, SAP30's high expression in basal/Claudin-low was shown to be significant compared to all other subtypes. Both PRKC and SAP30 gene expression exhibited a strong negative association with DNA methylation. Moreover, their expression varied between different stages and grades of the tumors. The higher expression of PRKCα was shown in stage I compared to stage II and in grade III compared to grade II. SAP30 expression was also higher in stage II than stage I and III and in grade III compared to grade I and II. Hypoxia enhanced the expression of Notch target genes HES1 and HEY1 in different breast cancer cells. This increase was more dramatic for HEY1 expression, suggesting that HEY1 may be a better marker of Notch activation in breast cancer cells [108]. While HES1 was shown to be highly expressed in the Luminal B subtype, a high expression level of HEY1 was found to be linked with the basal/claudin-low subtype with no significant differences when compared to the Luminal B subtype. Previously it has been clearly shown that CCND1 was amplified in breast cancer [111]. HES1 and CCND1 expression had a significant negative correlation with DNA methylation suggesting a potential epigenetic targeting therapeutic approach for these factors.

Human WNT2 is commonly upregulated in primary gastric and colorectal cancer and less frequently upregulated in primary breast cancer. The upregulation of WNT2 mRNA in breast cancer happens through estrogen [112, 113]. Loss of WNT5A has been associated with a poor prognosis in a various cancers, including breast and colon cancer, neuroblastoma, and leukemia, indicating that WNT5A might play a tumor-suppressive role in these malignancies [114]. In our results, when compared to other subtypes, basal/claudin-low showed a higher WNT2 expression and lowered WNT5A expression. Deregulation of Wnt/b-catenin signaling, including increased expression of Wnts and/or FZD and downregulation of FZD-related proteins, has been linked to breast cancer progression [115]. FZD5, a Frizzled family member, is preferentially expressed in TNBC and has a poor prognosis. Mechanistically, FZD5 contributed to TNBC cell G1/S transition, DNA replication/damage repair, survival, and stemness [116]. Downregulation of FZD7 in TNBC cell lines MDA-MB-231 and BT-20 deactivated Wnt signaling resulted in decreased cell proliferation and tumor transformation [117]. FZD1 high expression was found in the basal/claudin-low group as compared to HER2 and Luminal B. FZD5 high expression was significant in basal/claudin-low compared to other subtypes, FZD7 high expression was significant in both basal/claudin-low and normal groups compared to other subtypes.

In the current study, we chose Notch signaling pathway-related genes in order to determine their potential to be used as both therapeutic targets and prognostic markers. This is the first study that collected the TCGA expression data of all genes related to the Notch signaling pathway and provided a comprehensive view of their function in various breast cancer subtypes. We also provided a detailed overview of their relation to different stages and grades of breast cancer and their correlation to the survival rate in patients. This study makes it easier to design and introduce new targets as it determines each subtype’s specific expression pattern. Therefore, by targeting a combination of genes related to different levels of the Notch signaling pathway, it is possible to block cancer cell progression more efficiently, inhibit their ways of escaping from the immune system, and minimize the plausible resistance mechanisms with even fewer side effects.

In summary, we analyzed the expression of Notch-related pro-oncogenic factor genes in breast cancer subtypes and showed correlations between factor/receptor gene expression and patient tumor subtypes, tumor grades, and stages and survival and DNA methylation status using TCGA and METABRIC datasets. From the study, we found that WNT5A, ARRB1, HES1, JAG1, and HEYL are overexpressed in Luminal A; SKP1, APH1A, CCND1, FBXW11, KAT2A, RBX1, PSEN2, PSENEN are overexpressed in Luminal B; WNT2, NOTCH3, PPARD, DTX2, DTX4, LFNG are overexpressed in HER2 and, CUL1, SAP30, TCF7L2, ST3GAL6, PRKCA, NOTCH1, NOTCH2, MAML2, DLL1, DTX1, FZD1, FZD5, and FZD7 are overexpressed in basal breast cancer, suggesting their potential as therapeutic targets. Although our results indicate a correlation between the expressions of involved genes in the Notch signaling pathway and various subtypes, stages, grades, and DNA methylation of breast cancer, further screening studies of the Notch-related factor/receptor gene expression must be performed to validate the relationships within different subtypes due to the heterogenic primary tumor samples profiled by TCGA and METABRIC. These results can be applied as diagnostic or prognostic biomarkers and for treatment selection in breast cancer. The detailed carcinogenic mechanism of the Notch signaling elements in breast cancer is still unknown and also the exact functional impact of modulated NOTCH signaling remains particularly unclear and complex [118]. Considering the predominant oncogenic function of Notch signaling in breast cancer versus the tumor-suppressive role in Squamous cell carcinoma and other cancers suggests the intriguing dual role of this signaling pathway [82]. While these findings shed new light on the critical role of the Notch pathway in breast cancer pathogenesis, additional experimental investigations including analyzing different datasets and cohorts as well as post-translational modifications studies are required to characterize the precise mechanisms through which Notch signaling downstream or upstream regulators participate in breast cancer development. Multiple studies have reported different results regarding the Notch-related components expressions and their associations with various characteristics of study subjects including methylation status, subtypes, and tumor grade/stage status. In this study using gene expression profiling we found first that Notch genes activities are found to be context-dependent, leading to their playing differential roles within various subtypes. Secondly, according to the clear associations between mRNA expression and methylation status of several Notch-related components, epigenetic modification of the Notch pathway by promoter DNA methylation could be at least one of the primary mechanisms regulating the Notch pathway in breast cancer cells. Multiple studies have shown that non-coding RNAs are critical in breast cancer progression using different molecular mechanisms [119]. Also, differential expression of miRNAs and LncRNAs has been shown to play a significant role in many cancer types by regulating different pathways involved in tumorigenesis [120–123]. These non-coding RNAs regulate the Notch signaling pathway by interacting with the Notch-related genes at the transcriptional or posttranscriptional levels [32]. Accordingly, we conduct network analysis to find the relationship between miRNAs and LncRNAs with the Notch signaling-related mRNAs. Our findings based on bioinformatic analysis revealed five miRNAs and 10 LncRNAs interact with the Notch signaling-related genes in breast cancer (Fig. 7c, d, and e). Using the LncSEA database, the common Cancer-Hallmarks between LncRNA targets of miRNAs, which target mRNAs involved in the Notch signaling pathway, were performed. This analysis indicates that these LncRNAs are related to cancer hallmarks like EMT, metastasis, invasion, migration, apoptosis, and proliferation, previously shown as a critical hallmarks for breast cancer [124]. In the bioinformatic section, miR-16-5p was predicted as a possible target for Notch signaling pathways. This miRNA has been shown to suppress breast cancer proliferation [125]. Another study demonstrated that miR-16-5p has an inhibiting role in the Notch signaling pathway [126]. Therefore, it could be a diagnostic and prognostic potential biomarker in breast cancer. Altogether, modulating the expression levels of Notch pathway can be a potential therapeutic approach for breast cancer. As we outline herein, elucidating the novel prognostic and regulatory roles of Notch family and their function as essential mediators controlling breast cancer progression.

## Methods

### cBioPortal database analyses

Notch signaling effectors mRNA expression with molecular subtypes (PAM50) in patients with breast cancer were examined using METABRIC breast cancer cohort, the Molecular Taxonomy of Breast Cancer International Consortium [127]. The RNA-seq data (expression log intensity levels) from breast invasive carcinoma samples (n = 1904) and their relative clinical information are available at the TCGA data portal, cBioPortal for Cancer Genomics (http://www.cbioportal.org/). According to the PAM50 classification [128], METABRIC breast cancer patients were divided into 5 subtypes, including the Basal (n = 199), HER2 + (n = 220), Lum A (n = 679), Lum B (n = 461) and Normal-like (Normal) (n = 140). ER, PR and HER2 status were assessed using the patient’s IHC information.

### Correlation and Survival analyses

Pearson's correlation analysis was used to analyze of the correlation between the methylation degree and gene expression. The RNA-seq data and methylation data (Illumina Infinium Human Methylation 450) were downloaded from the Breast Invasive Carcinoma (TCGA, Firehose Legacy, https://www.cancer.gov/tcga). All samples that had undergone RNA sequencing and methylated chip data were used for correlation analysis of transcription and methylation. For pathway correlation analyses, the immune-evasion-related genes (genes up-regulated in highly immune-resistant cancer cells) comprised 20 genes obtained from the gene set LIN_TUMOR_ESCAPE_FROM_IMMUNE_ATTACK in The Molecular Signatures Database hallmark gene sets (MsigDB, software.broadinstitute.org/gsea/msigdb) [129, 130]. The metastasis-related genes comprised 29 genes obtained from the GILDEA_METASTASIS gene set [131]. Finally, the angiogenesis-related genes contained 21 genes obtained from the HU_ANGIOGENESIS_UP gene set [132]. Then mRNA expression data were extracted from METABRIC. Pearson's correlation analysis was used for the analysis of the correlation between genes. Kaplan–Meier overall survival analysis of breast cancer patients divided by the median expression level of Notch factors was performed in a platform for exploring Gene Expression patterns across Normal and Tumor tissues named GENT2 (http://gent2.appex.kr/gent2/) [133].

### GO and pathway analyses

To find the potential miRNAs that might target the studied mRNAs, we used miRNet (https://www.mirnet.ca/). miRNet is a web-based tool that uses several miRNA databases to find the predicted and experimentally validated target genes for miRNAs such as miRTarBase and TargetScan. By using miRNet, we recognized the predicted target LncRNAs of miRNAs. Then, the Sankey diagram for the mRNAs-miRNAs-LncRNAs network was drawn. Using the GeneMANIA prediction server -biological network integration for gene prioritization and predicting gene function- the protein–protein (PPI) networks performed for the genes involved in the Notch signaling pathway [134]. The FunRich v3.1.4 software was used to perform enrichment of genes in different cancer types using COSMIC data [135]. DIANA-miRPath v3.0 (http://www.microrna.gr/miRPathv3), an online bioinformatics tool, has been used to determine the KEGG molecular pathways and Gene Ontology (GO) that predicted miRNAs that might interact with the mRNAs involved in Notch signaling [136–138]. To determine the common Cancer-Hallmarks between LncRNA targets of miRNAs in this study, we performed enrichment analysis using the LncSEA database [139].

All the procedures were performed in accordance with the relevant guidelines and regulations.

## Supplementary Information

**Additional file 1.**

## Data Availability

All the raw data is available as below: TCGA (Breast Cancer (METABRIC, Nature 2012 & Nat Commun 2016)): https://www.cbioportal.org/study/summary?id=brca_metabric. TCGA (Breast Invasive Carcinoma (TCGA, Firehose Legacy)): https://www.cbioportal.org/study/summary?id=brca_tcga.
