# Supplementary material for: Notch signaling pathway: a comprehensive prognostic and gene expression profile analysis in breast cancer
Source: BMC Cancer. 2022 Dec 7;22:1282. doi: 10.1186/s12885-022-10383-z (PMC9730604; doi:10.1186/s12885-022-10383-z)
Supplement: Supplementary file 1 — Additional file 1. [file 12885_2022_10383_MOESM1_ESM.docx]

**Supplementary figure 1.**

**Differential expression of the NOTCH4, DLL3, and JAG2 in different breast cancer subtypes, grade, and stages.**


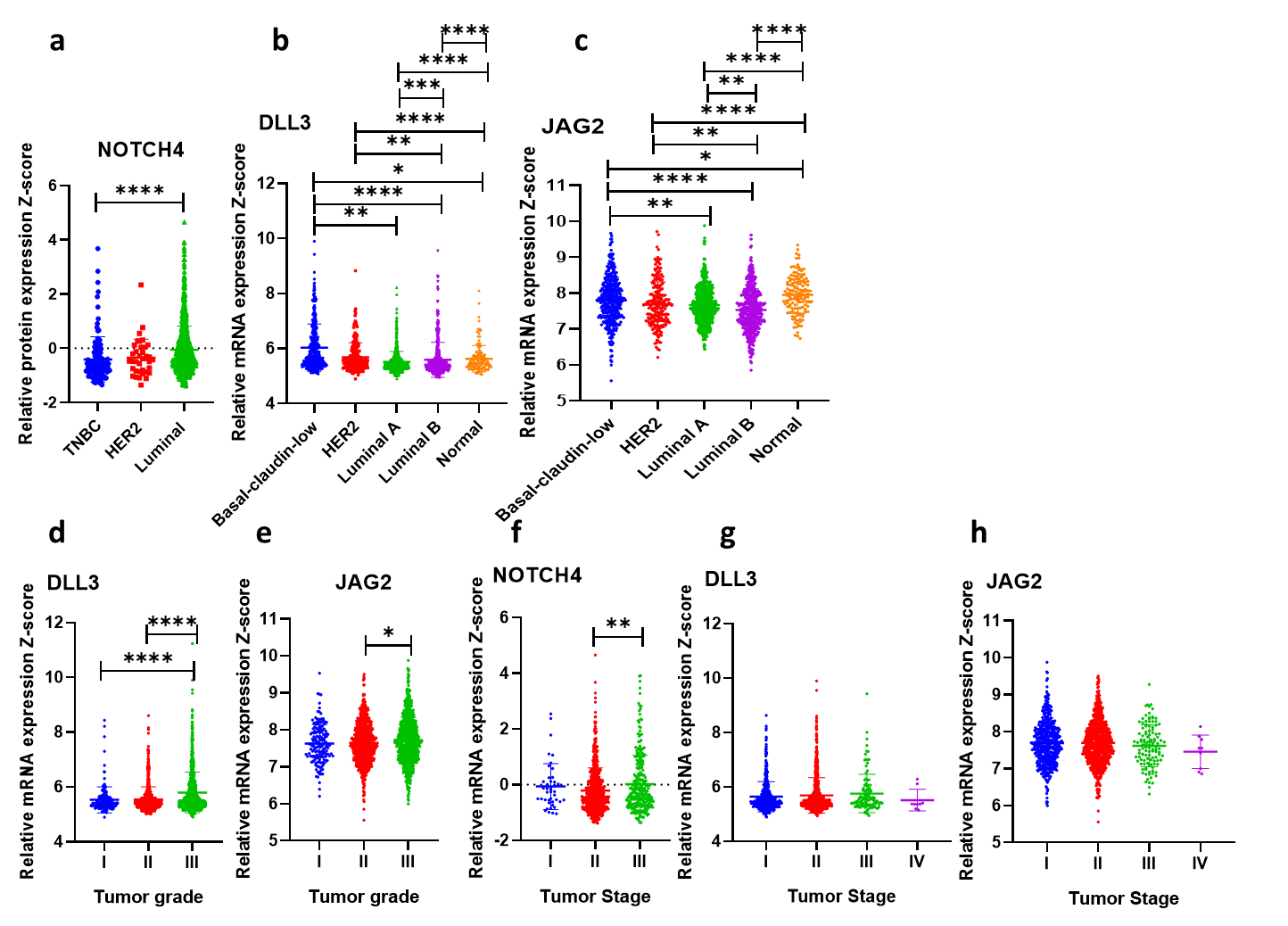


**Supplementary figure 1.**

**Differential expression of the NOTCH4, DLL3, and JAG2 in different breast cancer subtypes, grade, and stages. (a)** NOTCH4 (data from the TCGA, Firehose Legacy study), **(b, c)** DLL3, and JAG2 (data from the METABRIC study) gene expression in different breast cancer subtypes. **(d, e, f, g, and h)** Differential expression of the NOTCH4 (data from the TCGA, Firehose Legacy study), DLL3, and JAG2 (data from the METABRIC study) in different breast cancer grades and stages. Scatterplots show a significant association between breast cancer subtypes, grade, and stages. Data were analyzed by one-way ANOVA followed by Tukey’s post hoc test. Statistically significant values of (P-value*: P-value < 0.05, **: P-value < 0.001, ***: 0.0001 < P-value = 0.0001, ****: P-value < 0.0001) were determined.

**Supplementary figure 2.**

**Co-expression correlation between Notch signaling pathway elements and the Hedgehog, TGF-β and AKT signaling pathways.**

**
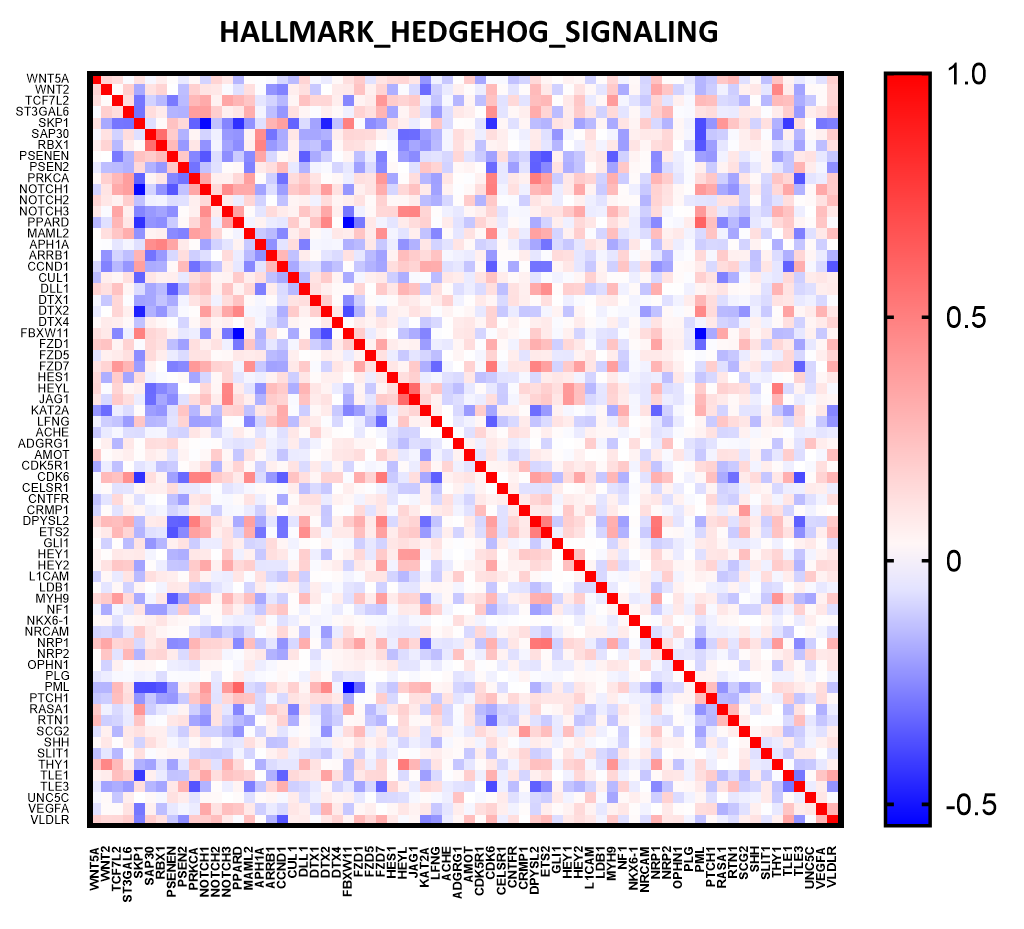
**

**Supplementary figure 2. A**

**
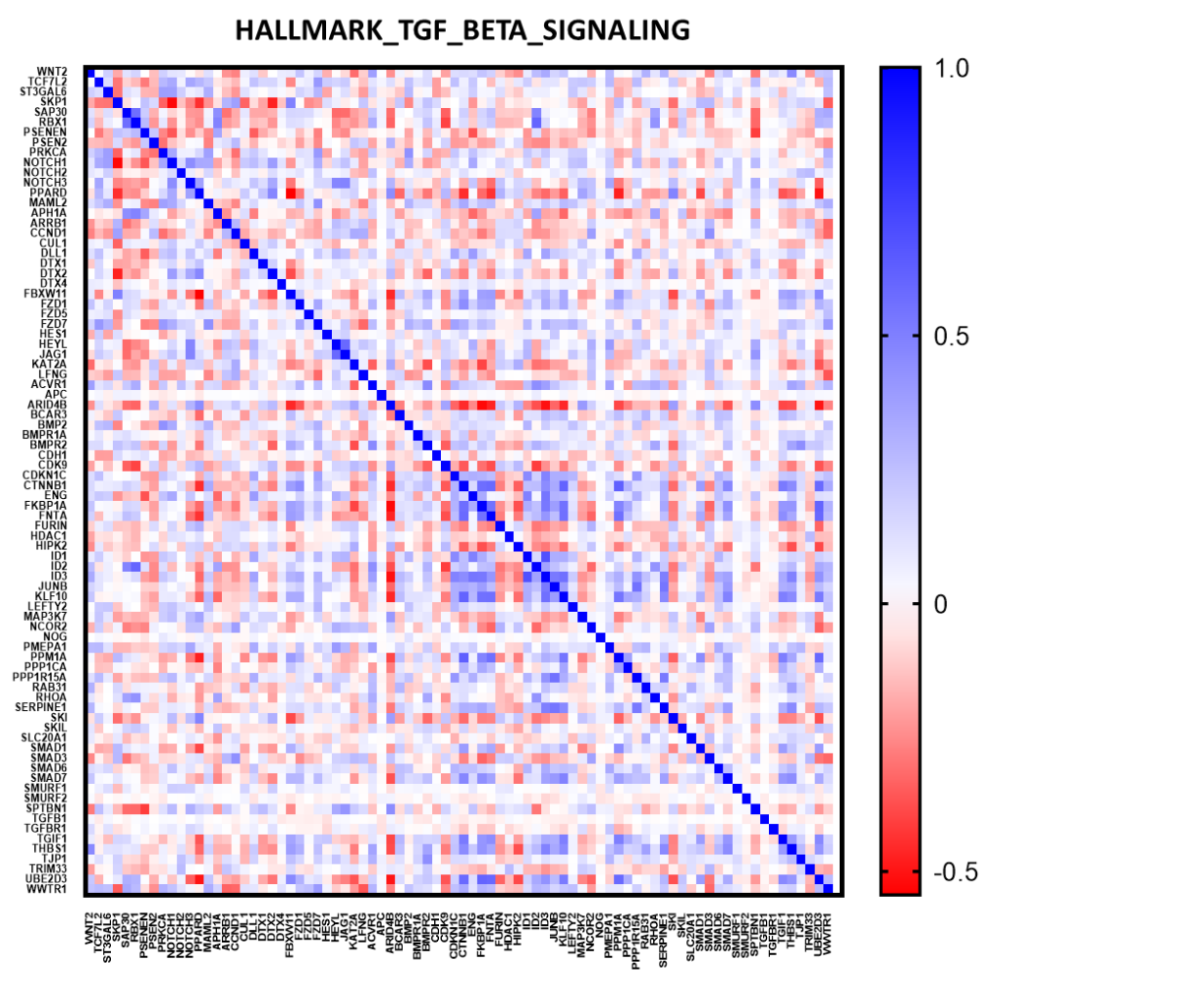
**

**Supplementary figure 2. b**

**
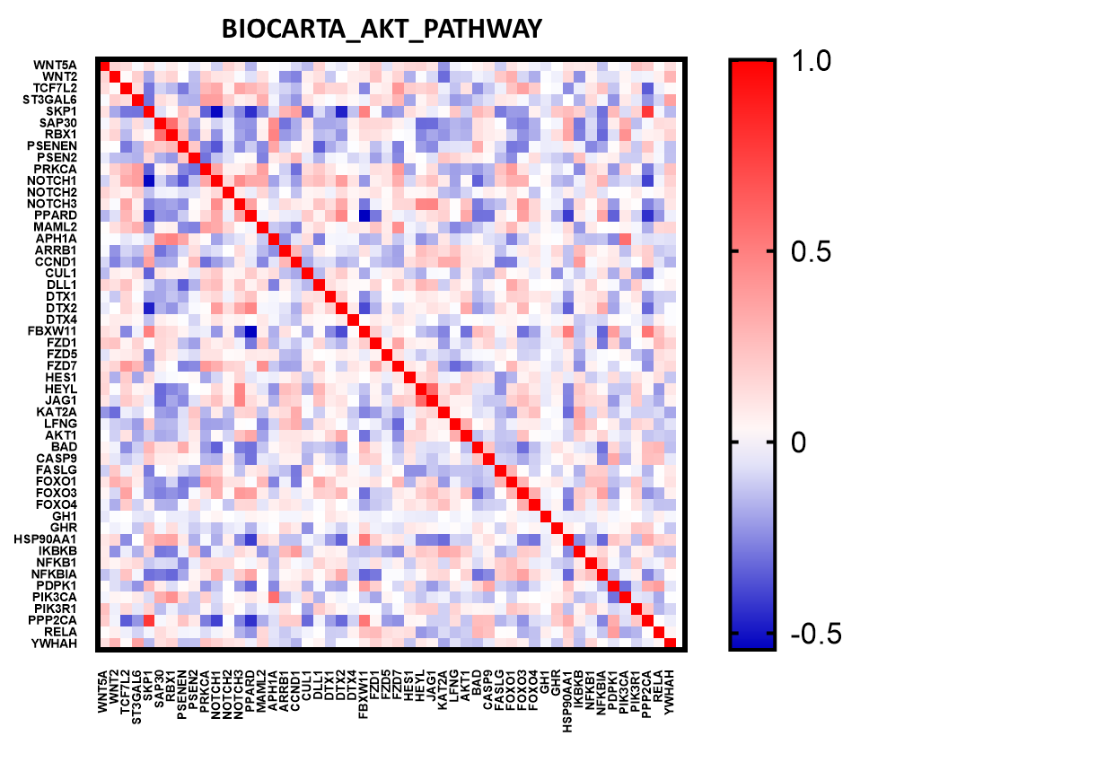
**

**Supplementary figure 2. c**

**Supplementary figure 2.** **Co-expression correlation between Notch signaling pathway elements and the Hedgehog, TGF-β and AKT signaling pathways.** Co-expression (mRNA) correlation analysis between Notch pathway elements and Hedgehog , TGF-β and AKT signaling pathways markers using **(a)**, HALLMARK_HEDGEHOG_SIGNALING **(b)**, HALLMARK_TGF_BETA_SIGNALING, and **(c)**, BIOCARTA_AKT_PATHWAY gene sets, respectively ([https://www.gsea-msigdb.org/gsea/msigdb](file:///C:\Users\hassa\Desktop\Notch%20paper\abstract)). Correlation heatmap (Pearson r) of the transcriptomes from METABRIC breast cancer project samples (n= 1986). The red color refers to negative correlation, and the blue color indicates positive correlation. The mRNA expression data were extracted from METABRIC. Pearson's correlation analysis was used for the analysis of co-expression between genes.

**Supplementary figure 3.**

**Kaplan–Meier online platform (kmplot.com) validated the association of clinical outcome for several Notch-regulating factors based on relapse-free survival (RSF) using PAM50 classification for TNBC subtype.**


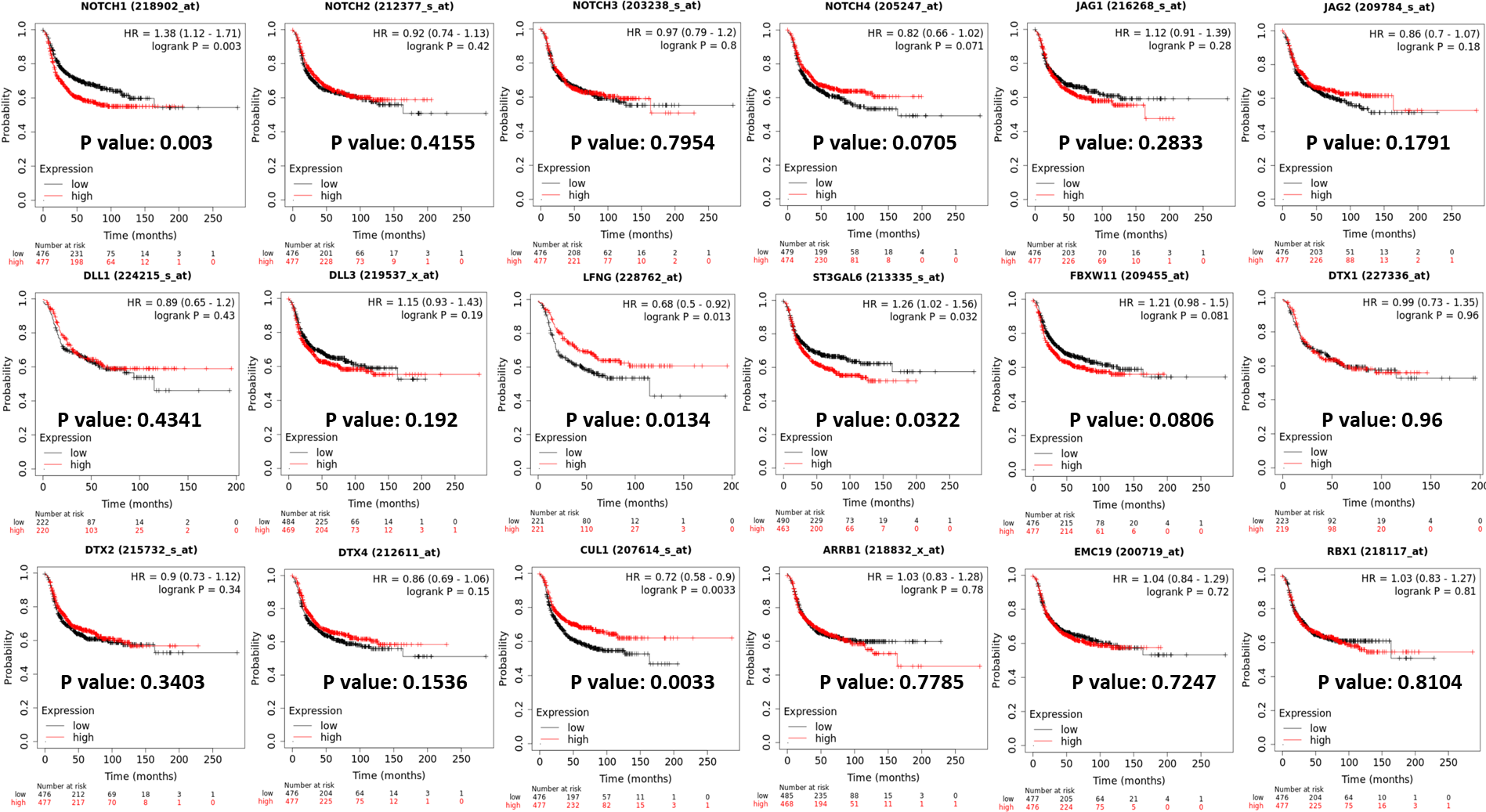


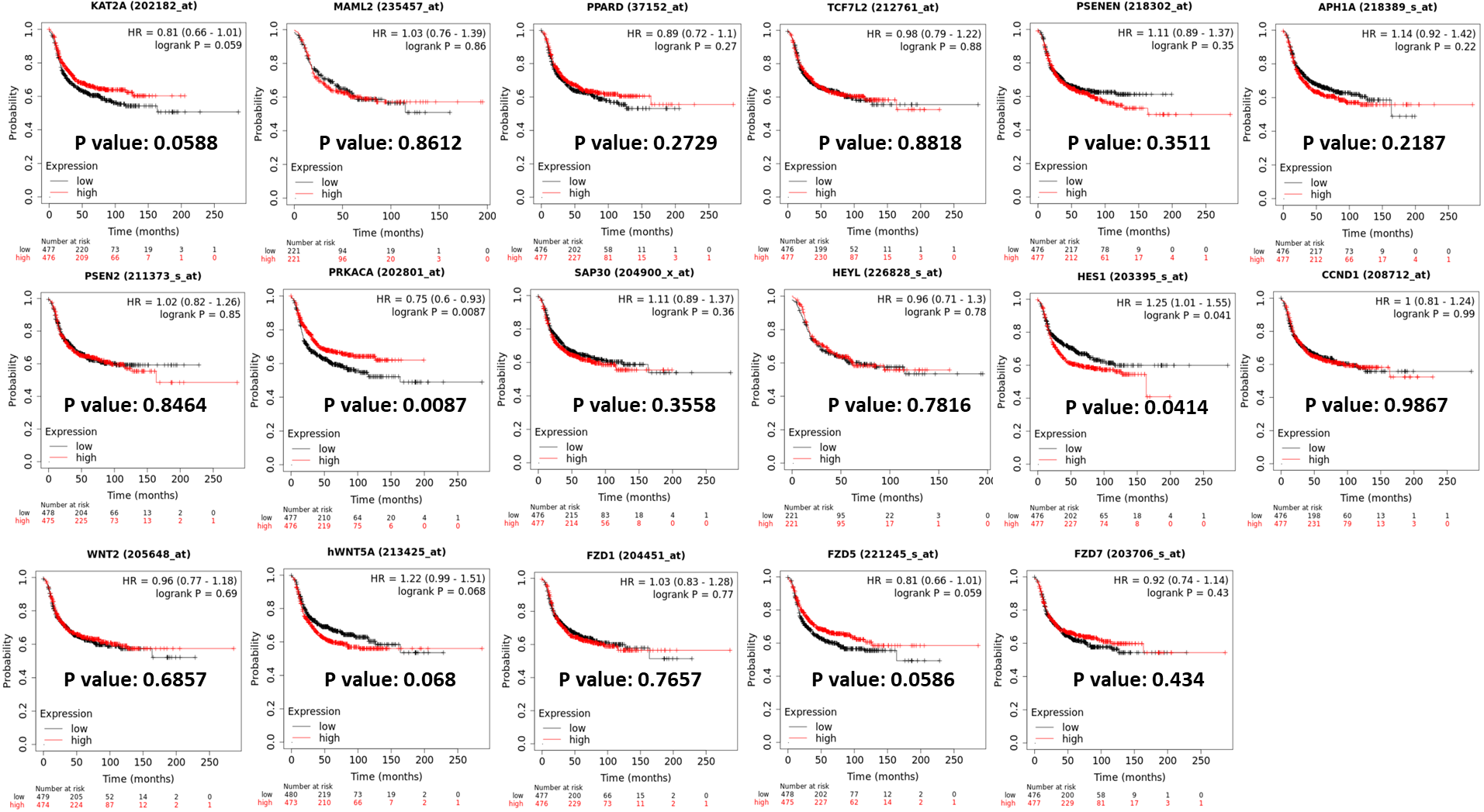
**Supplementary figure 3a.**  **Kaplan–Meier online platform (kmplot.com) validated the association of clinical outcome for several Notch-regulating factors based on relapse-free survival (RSF) using PAM50 classification for TNBC subtype.** Kaplan-Meier analysis of RFS was used based on the mean value of gene in breast cancer.

**Supplementary figure 3b.**  **Kaplan–Meier online platform (kmplot.com) validated the association of clinical outcome for several Notch-regulating factors based on relapse-free survival (RSF) using PAM50 classification for TNBC subtype.** Kaplan-Meier analysis of RFS was used based on the mean value of gene in breast cancer.
